# Ferroptosis in Septic Cardiomyopathy Is Alleviated by Ondansetron: The Critical Role of the HTR3A-ATF3 Axis in Mitochondrial and Oxidative Homeostasis

**DOI:** 10.3390/biomedicines14051040

**Published:** 2026-05-03

**Authors:** Xinyun Wang, Yangyi Lin, Wei Liu, Yufeng Wu, Boshen Yang, Yiming Qi, Yipeng Zhang, Yuanyuan Jin, Yuanlong Wang, Kaifan Niu, Xian Jin

**Affiliations:** 1 Department of Cardiology, Shanghai Sixth People’s Hospital, Shanghai Jiao Tong University School of Medicine, Shanghai 200233, China; 2 International Medical College of Chongqing Medical University, Chongqing 400010, China; 3 Department of Cardiology, Tongren Hospital, Shanghai Jiao Tong University School of Medicine, Shanghai 200050, China

**Keywords:** ondansetron, ferroptosis, septic cardiomyopathy, *ATF3*, mitochondrial dysfunction, oxidative stress

## Abstract

**Background:** Emerging evidence has established ferroptosis as a vital factor in the pathogenesis of cardiovascular diseases, especially in septic cardiomyopathy (SCM). Meanwhile, ondansetron (OND), a well-established 5-HT3 receptor antagonist, has gained increasing attention for its pleiotropic effects. However, its potential to modulate ferroptosis in the cardiovascular field remains unexplored. This study aims to fill this gap by exploring the potential of OND as an innovative therapeutic intervention for SCM. **Methods:** This study utilized both in vitro and in vivo models of septic cardiomyopathy (SCM), which was induced by lipopolysaccharide (LPS) stimulation in neonatal rat cardiomyocytes (NRCMs) and C57BL/6 mice. Through RNA sequencing, as well as molecular and functional assessments—including echocardiography and ferroptosis-related measurements—we revealed the anti-ferroptotic effect of ondansetron (OND). Mechanistically, *ATF3* was identified as a pivotal regulator, with its overexpression via AAV9 in vivo and ADV in vitro confirming its role in OND-induced cardioprotection. **Results:** Ondansetron (OND) showed potent anti-ferroptotic effects in both cellular and murine models of septic cardiomyopathy (SCM). Treatment with OND not only improved cardiac performance but also reduced ferroptotic markers, mitigated lipid peroxidation and iron overload, and bolstered antioxidant defense. Notably, OND administration attenuated oxidative and endoplasmic reticulum (ER) stress while restoring mitochondrial integrity. Mechanistically, the anti-ferroptotic activity of OND was mediated through the *HTR3A/ATF3* axis: ATF3 overexpression negated OND’s protective effects, while HTR3A antagonism with VUF10166 recapitulated its benefits. Conversely, HTR3A agonism with PBG attenuated ferroptosis resistance, further implicating this pathway as central to OND’s mechanism. **Conclusions:** This study demonstrated a novel pharmacological role for ondansetron (OND) in attenuating ferroptosis in septic cardiomyopathy (SCM) via the *HTR3A/ATF3* signaling pathway. This finding delineates a novel therapeutic avenue and supports the repurposing of OND beyond its traditional antiemetic use to cardiovascular applications.

## 1. Introduction

Septic cardiomyopathy (SCM) is a serious and frequent early complication of sepsis, occurring in 18% to 65% of septic patients, and is responsible for mortality rates of 36% to 55% [1]. Pathologically, it is defined by reversible biventricular dilation with severely impaired left ventricular contractility in the context of systemic infection [2]. Notwithstanding its high frequency and high mortality rate, the existing treatment for SCM is still purely supportive, focusing on anti-infection therapy, fluid management, and support of organ function, with no specific pharmacologic therapies available [3]. Thus, the search for effective modalities for the prevention and early management of sepsis-induced cardiac injury is a pressing clinical need.

However, recent studies have revealed a strong link between ferroptosis and the development of sepsis-induced cardiomyopathy (SCM) [4,5]. Ferroptosis is a novel type of regulated cell death; it is iron-dependent and involves lipid peroxidation, initiated by the inactivation of glutathione peroxidase 4 (GPX4), depletion of glutathione (GSH), and Fenton reactions mediated by Fe^2+^, resulting in the peroxidation of membrane-bound polyunsaturated fatty acids (PUFAs) [6]. To further support the involvement of ferroptosis in SCM, the ferroptosis inhibitor Ferrostatin-1 has been demonstrated to inhibit chemokine signaling, attenuate neutrophil infiltration, and greatly enhance cardiac function in mice with CLP-induced sepsis [7]. Collectively, these observations emphasize the crucial role of ferroptosis in the progression of SCM and its potential as a novel therapeutic target for the improvement of patient outcomes.

Oxidative stress and mitochondrial dysfunction are the intersecting pathological mechanisms underlying both ferroptosis and septic cardiomyopathy (SCM). The mtROS overproduction induced by sepsis contributes to mitochondrial structural damage, thus rendering cells susceptible to ferroptosis-induced death [8]. Conversely, the progression of ferroptosis further impairs mitochondrial respiratory activity [9]. The importance of maintaining mitochondrial integrity is further underscored by the observation that the multifaceted dysfunction of this organelle, including ROS accumulation, depolarization, and dynamic abnormalities, contributes to myocardial bioenergetic dysfunction and systolic dysfunction in SCM [10]. These observations collectively suggest that the therapeutic approach targeting the attenuation of oxidative stress and the preservation of mitochondrial function may offer a common strategy against SCM pathology and ferroptotic cell death.

Ondansetron (OND) is traditionally used as a 5-HT3 receptor antagonist for the treatment of nausea and vomiting [11]. However, recent studies have found that it has wider pharmacological effects, including anti-inflammatory effects. In particular, Ondansetron has been found to inhibit the activation of peritoneal macrophages and the expression of pro-inflammatory cytokines (IL-1β, IL-6, and TNF-α) and iNOS in a murine intestinal model of SCM [12], as well as alleviate pancreatic injury in acute pancreatitis [13]. These findings led us to explore whether ondansetron might have therapeutic potential in septic cardiomyopathy due to its anti-inflammatory properties. However, the relationship between ondansetron and ferroptosis, a core pathogenic mechanism in SCM, has not been clarified.

This research aimed to explore whether ondansetron has a therapeutic role in septic cardiomyopathy (SCM), and its possible link with ferroptosis, as well as the underlying molecular mechanisms. We found that ondansetron suppressed cardiomyocyte ferroptosis in septic cardiomyopathy, thus relieving sepsis-induced cardiac injury. Moreover, OND relieved oxidative injury and mitochondrial damage and strongly suppressed cardiomyocyte ferroptosis. Mechanistically, this is achieved through HTR3A receptor antagonism, resulting in ATF3 downregulation and subsequent normalization of the anti-ferroptotic factors SLC7A11 and GPX4. Our results indicate that OND can be repurposed as a potential therapeutic drug for SCM by targeting the *HTR3A/ATF3* pathway.

## 2. Materials and Methods

**Reagents**

Ondansetron powder (purity 99.37%, MedChemExpress, HY-B0002B, Monmouth Junction, NJ, USA) was dissolved in dimethyl sulfoxide (DMSO; Solarbio, D8371-50, Beijing, China) to prepare a 10 mg/mL (34.09 mM) stock solution. For in vitro experiments, the stock solution was diluted with Dulbecco’s Modified Eagle Medium (DMEM); for in vivo studies, it was diluted with corn oil (Beyotime, ST2308, Shanghai, China). Lipopolysaccharide (LPS; Sigma-Aldrich, L2880, St. Louis, MO, USA) was dissolved in saline prior to use. VUF 10166 (Selleck Chemicals, S2865, Houston, TX, USA), 1-phenylbiguanide (PBG; Selleck Chemicals, S2578, Houston, TX, USA) and ferrostatin-1(Fer-1; Selleck Chemicals, S7243, Houston, TX, USA) were obtained commercially. All reagents were stored and used according to the manufacturer’s instructions.

**Cell culture and treatment**

For the isolation of neonatal rat cardiomyocytes (NRCMs), hearts were removed from 1- to 3-day-old Sprague Dawley rat pups after euthanasia. After perfusion with phosphate-buffered saline (PBS) to clean blood cells, the cardiac tissue was removed and digested with collagenase II for 4–6 h at 4 °C. The resulting cell suspension was centrifuged, resuspended, and processed through a differential adhesion technique to enrich cardiomyocytes. To further suppress the proliferation of contaminating non-myocytes, bromodeoxyuridine (BrdU; Beyotime, SH0136, Shanghai, China) was added to the culture medium. The purified NRCMs were subsequently maintained in Dulbecco’s Modified Eagle Medium (DMEM) supplemented with 10% fetal bovine serum (FBS) and 1% penicillin–streptomycin.

To ascertain a safe and effective dosage, a CCK-8 assay was initially performed, which demonstrated that OND concentrations up to 100 μM did not significantly affect NRCM viability (Figure S3). Therefore, 25 μM and 75 μM of OND were chosen to intervene in the LPS-induced (20 μg/mL, 12 h) in vitro SCM model [14,15]. OND treatment was initiated one hour after LPS stimulation (20 μg/mL, 12 h) in NRCMs, yielding four experimental groups: control, LPS, LPS + OND (25 μM), and LPS + OND (75 μM). The 5-HT3 receptor antagonist VUF 10166 (0.1 μM) [16,17] and the 5-HT3 receptor agonist 1-phenylbiguanide (PBG)(50 μM) [18,19] were also added 1 h after LPS stimulation. Ferrostatin-1(Fer-1) (2 μM) [20] was added 12 h before LPS stimulation. The dose of the drugs was chosen according to the literature and the manufacturer’s guide.

**Animal models and experimental procedures**

Preclinical research on septic cardiomyopathy commonly employs two established animal models: the LPS-induced endotoxemia model and the cecal ligation and puncture (CLP) model [21,22]. The majority of the in vivo experiments conducted were based on the LPS-induced endotoxemia model. To establish the LPS endotoxemia model of SCM mice, 6- to 8-week-old male C57BL/6 mice were administered a single i.p. injection of LPS (10 mg/kg). Each mouse weighed approximately 20 to 25 g. One hour later, mice were administered the HTR3A antagonist ondansetron (0.25 or 1 mg/kg), the HTR3A antagonist VUF 10166 (0.1 mg/kg), or the HTR3A agonist PBG (1 mg/kg), according to the previously published literature [16,23,24]. Control mice received DMSO or corn oil alone. All mice were sacrificed 12 h post-LPS injection.

The cecal ligation and puncture (CLP) procedure was employed to induce septic cardiomyopathy in mice. Briefly, mice were anesthetized with isoflurane inhalation, and a small midline laparotomy was performed to expose the cecum. The cecum was ligated below the ileocecal valve, and the ligated distal tip was punctured once through-and-through with a 21-gauge needle. A small amount of fecal content was gently extruded to ensure patency of the puncture site. The cecum was then returned to the peritoneal cavity, and the abdominal incision was closed in layers. Animals were then allowed to recover from anesthesia. Sham-operated controls underwent laparotomy and cecal manipulation without ligation or puncture. OND was administered at the time of CLP induction. At 24 h post-operation, mice were subjected to echocardiography and subsequent tissue harvesting.

All procedures were conducted in the specialized animal surgery facility. Mice were acclimatized for a week prior to experiments, and housed under standard conditions (22 ± 2 °C, 12 h light/dark cycle, free access to food and water). All surgeries were performed under isoflurane anesthesia (2% for induction, 1.5% for maintenance). Euthanasia was performed under deep anesthesia through cervical dislocation.

Each individual mouse was considered an independent experimental unit. The mice were randomly allocated into 8 groups (Control, LPS, LPS + OND0.25 (0.25 mg/kg), LPS + OND1 (1 mg/kg), ATF3 + LPS, ATF3 + LPS + OND, LPS + VUF10166, LPS + OND + PBG) with 6–8 animals per group. The total number of experimental animals was less than 60. Our preliminary experimental data indicated that this group size was sufficient to achieve consistent results and account for biological variability while minimizing the total number of animals used in accordance with ethical guidelines.

All the inclusion and exclusion criteria for animals and data points were set before the initiation of the experiment. Animals were included if they successfully underwent the surgical procedure without complications. Exclusion criteria (predefined) included: death during surgery, failure to complete the experimental protocol, or technical issues with sample collection. Apart from those that met the exclusion criteria, no animals, experimental units, or data points were excluded from the analysis in any group. All collected data were included in the final statistical analysis.

Randomization was used to allocate experimental units to the control and treatment groups. A simple random sampling method was employed using a random number table generated by statistical software (IBM SPSS Statistics 27.0). To minimize potential confounders, all animals were housed under identical conditions with randomized cage locations. The order of treatments and measurements was also randomized. Outcome assessors and data analysts were blinded to group allocation.

**Adenovirus (Adv) and Adeno-Associated Virus 9 (AAV9) Transfection**

Adenovirus (Adv) and adeno-associated virus (AAV) vectors were packaged by Genechem (Shanghai, China) and WZ Biosciences Inc. (Jinan, China). For cardiomyocyte-specific overexpression, ATF3 was manipulated with adenoviral (Adv) and adeno-associated viral (AAV) vectors. In vitro experiments were performed using neonatal rat cardiomyocytes (NRCMs), which were incubated in serum-free medium for half an hour before infection with Adv-HA or Adv-HA-ATF3 (MOI = 50) for 6 h. After 48 h of further culture in fresh DMEM with 10% FBS, the cells were harvested for subsequent experiments. In vivo, to achieve cardiomyocyte-specific overexpression of ATF3, we employed AAV9 vectors driven by the cardiac troponin T (cTnT) promoter. Mice received a tail vein injection of either AAV9-cTnT-ATF3 (4 × 10^11^ v.g./mouse) or the control AAV9-cTnT vector. After a 4-week period, heart tissues were collected for further analysis.

**Western blotting**

Protein lysates from cells or myocardial tissues (RIPA buffer, P0013B, Beyotime, Shanghai, China) were normalized to 1 µg/µL after centrifugation; 10 ug per sample was resolved by SDS-PAGE, transferred to PVDF membranes (WGPVDF45,Servicebio, Beijing, China), blocked, and probed overnight at 4 °C with primary antibodies (Table S1), followed by HRP-conjugated secondary antibodies (Beyotime, Shanghai, China) for 1 h at 25 °C. Immunoreactive bands were detected by ECL (Tanon, Shanghai, China) and quantified with ImageJ (v1.53t); β-actin was used as a loading control.

**Real-Time PCR**

Total RNA was taken from heart tissue or cells using an RNA isolation kit (#R701, Vazyme, Nanjing, China). It was then turned into cDNA with HiScript II QRT SuperMix (R223-01, Vazyme, Nanjing, China). We used AceQ Universal SYBR qPCR Master Mix (#Q511, Vazyme, Nanjing, China) for RT-qPCR on a LightCycler 96 (Roche, Indianapolis, IN, USA). We compared the cDNA expression to that of HPRT. Primer details are presented in Tables S2 and S3.

**Immunofluorescence**

For immunofluorescence, heart sections were deparaffinized, rehydrated, and processed for antigen retrieval (citrate buffer). Cells were washed with PBS and fixed with 4% paraformaldehyde. All samples were permeabilized with 0.1% Triton X-100, blocked with 3% BSA, and probed with primary antibodies (Table S4) and fluorescent secondary antibodies. Nuclei were counterstained with DAPI. Images were taken with confocal microscopy (Zeiss, Oberkochen, Germany), and fluorescence intensity was measured by using ImageJ (v1.53t).

**Histological Examination**

Mouse hearts were fixed in 4% buffered paraformaldehyde, embedded in paraffin, and cut into 5 μm sections. For histopathology, sections were deparaffinized, rehydrated, and stained with hematoxylin and eosin (HE) using standard methods. Prussian blue staining was done with a commercial kit (G1428, Solarbio, Beijing, China) following the manufacturer’s instructions.

**Transmission electron microscopy (TEM)**

For ultrastructural studies, NRCMs were collected and spun down. Samples were washed with PBS, incubated overnight in 2.5% glutaraldehyde at 4 °C, and subsequently fixed with 1% osmium tetroxide at 4 °C for 2 h. After that, samples were dehydrated with ethanol (50%, 70%, and 90%), then a mix of 90% ethanol and 90% acetone, and finally pure acetone. They were infiltrated with acetone–resin mixtures in ratios of 1:1, 1:2, and 1:3 for 1 h each, and a 1:3 mixture overnight. The solution was replaced with pure resin for 3 h, and this was repeated once. Resin was hardened at 60 °C for 48 h. Ultrathin sections (60–80 nm) were made, stained with uranyl acetate and lead citrate, and viewed with a Hitachi HT7800 transmission electron microscope (Hitachi High-Technologies, Tokyo, Japan) to study mitochondrial structure.

**Procedures for RNA sequencing (RNA-seq) and data analysis**

The total RNA obtained from six samples of myocardial tissue, representing three biological replicates per group (LPS + DMSO and LPS + OND 1 mg/kg), was analyzed for RNA sequencing by Liebing Biomedical Technology Co., Ltd. (Shanghai, China). The analysis of the data was done through the cloud-based platform of the company using the DESeq2 algorithm (v.1.52.0, Bioconductor). The differentially expressed genes (DEGs) were identified by a threshold of fold change >1.5 or <0.667, with a *p*-value and false discovery rate (FDR) both <0.05. Gene Ontology (GO) enrichment analysis was done using the NCBI, UniProt, and Gene Ontology resources, with significance determined by Fisher’s exact test (*p*-value < 0.05). The KEGG pathway analysis was also done using Fisher’s exact test (*p*-value < 0.05).

**Echocardiography**

Based on earlier findings that heart function in LPS-induced mouse models of septic cardiomyopathy (SCM) is lowest at 12 h after injection, this time was chosen for functional assessment in this study [25,26]. Mice were anesthetized with 1.5% isoflurane in 0.7 L/min oxygen and placed on their backs. Two-dimensional B-mode images in the parasternal long-axis view were used to guide M-mode recordings for the papillary muscles. The left ventricle’s systolic function was measured by calculating the ejection fraction (EF%) and fractional shortening (FS%). To reduce variability, these values were averaged over at least five consecutive heartbeats.

**siRNA Transfection**

The siRNA targeting HTR3A (Sense Strand: 5′-GGUGCAUAAGCAGGAUUUATT-3′, Antisense Strand: 5′-UAAAUCCUGCUUAUGCACCTT-3′), siRNA targeting ATF3 (Sense Strand: 5′-AGCCUGGUGUUGUGCUAUUUATT-3′, Antisense Strand: 5′-UAAAUAGCACAACACCAGGCUTT-3′), and scrambled siRNA control (Sense Strand: 5′-UUCUCCGAACGUGUCACGU-3′, Antisense Strand: 5′-ACGUGACACGUUCGGAGAA-3′) were obtained from GenCefe Bio (Wuxi, China). siRNA transfection was done using an RNAi Reagent (CALNP, D-Nano Medical, Beijing, China).

**Detection of ROS levels**

The measurement of ROS in NRCMs was performed using DCFH-DA (S0033S, Beyotime, Shanghai, China) for intracellular ROS and using MitoSOX Red (HY-D1055, MedChemExpress, Monmouth Junction, NJ, USA) for mitochondrial ROS. Cells were plated in 12-well plates at about 50% confluence and stained following the manufacturer’s protocol, and the fluorescence intensity was determined using a fluorescence microscope (TH4-200, OLYMPUS, Tokyo, Japan).

**Detection of Glutathione (GSH) and Malondialdehyde (MDA) levels**

The levels of malondialdehyde (MDA) and glutathione (GSH) were determined in cell and tissue samples using commercial kits (BC0020 and BC1175, Solarbio, Beijing, China).

**Cell viability detection**

We assessed cell viability using the Calcein-AM/PI double staining kit (C2015S, Beyotime, Shanghai, China). Fluorescence microscopy images were taken, and the live/dead cell ratio (calcein/PI fluorescence intensity) and mean fluorescence intensity of each group were analyzed by ImageJ.

**Measurement of mitochondrial membrane potential**

JC-1 staining (M8650, Solarbio, Beijing, China) was used to evaluate MMP. After staining, fluorescence images were taken, and the red/green fluorescence ratio or mean intensity for each group was analyzed using ImageJ for statistical analysis.

**Bodipy 493/503 and Bodipy 581/591 C11 staining**

Lipid droplet accumulation and lipid peroxidation in cells were analyzed using Bodipy493/503 and Bodipy581/591 C11 probes (C2053S and S0043S, Beyotime, Shanghai, China) respectively. Fluorescence images were taken, and ImageJ was used to analyze the relative and mean fluorescence intensities for further statistical analysis.

**Detection of Fe2+ levels**

Intracellular Fe^2+^ concentration was measured by the fluorescent probe FerroOrange (G1727-100T, Servicebio, Wuhan, China), and fluorescence images were analyzed to determine signal intensity.

**Statistics**

All statistical analyses were performed using the GraphPad Prism software (version 9.5.3). Two-group comparisons were done using the unpaired Student’s *t*-test. For multiple comparisons, one-way or two-way ANOVA was used, followed by Tukey’s multiple comparisons test if the data followed a normal distribution; otherwise, the non-parametric Kruskal–Wallis test with Dunn’s post hoc test was used. A *p*-value of less than 0.05 was considered statistically significant. The data are expressed as the mean ± standard deviation. * *p* < 0.05, ** *p* < 0.01, *** *p* < 0.001, **** *p* < 0.0001, ns, not significant. Data were obtained from at least *N* = 3 independent biological replicates (defined as three independent cell preparations) for in vitro experiments and from at least *N* = 3 independent biological replicates (defined as three independent animal samples) for in vivo experiments; all experiments were independently repeated at least three times.

## 3. Results

### 3.1. LPS-Induced Ferroptosis in NRCM Cells Is Alleviated by Ondansetron

**Figure 1:**
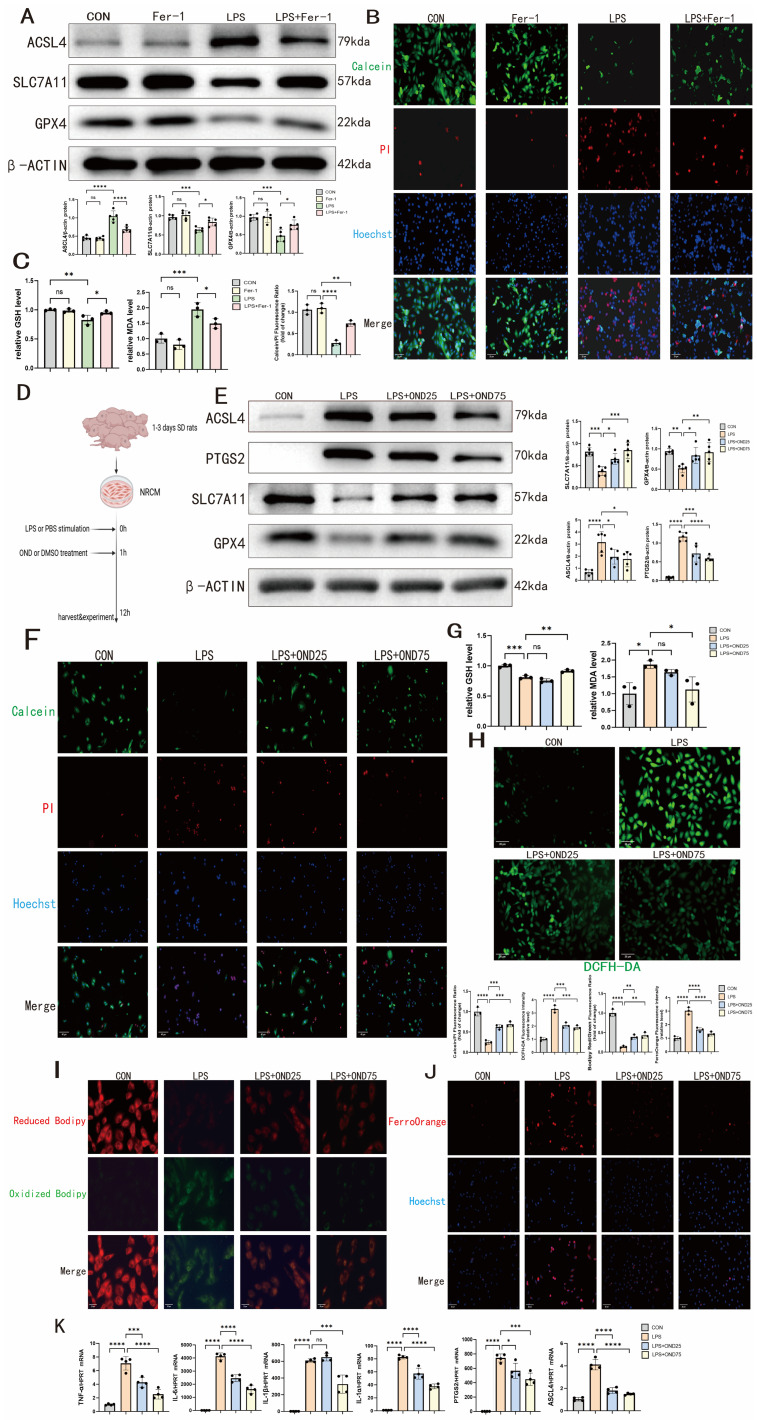
Ondansetron suppresses ferroptosis in LPS-stimulated NRCMs. (**A**) Representative Western blotting bands and quantification of ACSL4, SLC7A11, and GPX4 protein levels in NRCMs exposed to LPS with or without ferrostatin-1 (Fer-1) treatment. *N* = 5. (**B**) Representative fluorescence micrographs and quantitative assessment of Calcein-AM/PI and Hoechst staining in LPS-challenged NRCMs following Fer-1 administration. Scale bar = 20 μm. *N* = 3. Green indicates Calcein-AM (live cells), red indicates PI (dead cells), and blue indicates Hoechst (nuclei). (**C**) Relative intracellular glutathione (GSH) content and malondialdehyde (MDA) levels in LPS-stimulated NRCMs treated with Fer-1. *N* = 3 (**D**) Schematic diagram of the NRCM in vitro experiment. (**E**) Western blotting bands and statistical graphs of their representative ferroptosis markers, including ACSL4, PTGS2, SLC7A11, and GPX4, after LPS-stimulated NRCMs without or after treatment with different concentrations of OND (25 μM, 75 μM). *N* = 5. (**F**) Representative images and quantitative analysis of Calcein/PI and Hoechst staining after LPS-stimulated NRCMs without or after treatment with different concentrations of OND (25 μM, 75 μM). Scale bar = 40 μm, *N* = 3. Green indicates Calcein-AM (live cells), red indicates PI (dead cells), and blue indicates Hoechst (nuclei). (**G**) The relative GSH and MDA contents detected after NRCM were stimulated by LPS, without or after treatment with different concentrations of OND (25 μM, 75 μM). *N* = 3. (**H**) Representative images and quantitative analysis of DCFH-DA staining after LPS-stimulated NRCM without or after treatment with different concentrations of OND (25 μM, 75 μM). Scale bar = 20 μm, *N* = 3. Green indicates DCFH-DA (ROS) (**I**) Representative images and quantitative analysis of Bodipy581/591C11 staining after NRCM stimulated by LPS without or with different concentrations of OND (25 μM, 75 μM). Scale bar = 20 μm, *N* = 3. **Red fluorescence indicates Reduced Bodipy, and green indicates Oxidized Bodipy (lipid peroxidation)**. (**J**) Representative images and quantitative analysis of FerroOrange and Hoechst staining after NRCM stimulated by LPS without or with different concentrations of OND (25 μM, 75 μM). Scale bar = 20 μm, *N* = 3. Red indicates FerroOrange (Fe^2+^), blue indicates Hoechst (nuclei). (**K**) After NRCMs were stimulated with LPS and treated with or without different concentrations of OND (25 μM or 75 μM), qRT-PCR was used to detect mRNA levels of inflammatory factors and ferroptosis markers, including IL-1α, IL-1β, IL-6, TNF-α, PTGS2, and ACSL4. *N* = 4. For multiple comparisons, one-way or two-way ANOVA was used, followed by Tukey’s multiple comparisons test. A *p*-value of less than 0.05 was considered statistically significant. The data are expressed as the mean ± standard deviation. * *p* < 0.05, ** *p* < 0.01, *** *p* < 0.001, **** *p* < 0.0001, ns, not significant. Data were obtained from independent biological experiments. Black dots represent individual data points.

To establish the involvement of ferroptosis in septic cardiomyopathy, we first employed the ferroptosis inhibitor ferrostatin-1 (Fer-1) in an in vitro model of LPS-stimulated NRCMs. As illustrated in Figure 1A, LPS exposure resulted in pronounced upregulation of ACSL4 and concomitant downregulation of SLC7A11 and GPX4 at the protein level, both of which were partially reversed by Fer-1 treatment. LPS-induced cell death was also alleviated by Fer-1, as reflected by increased Calcein retention and reduced PI uptake (Figure 1B). Additionally, Fer-1 attenuated the LPS-triggered decline in GSH content and elevation in MDA levels (Figure 1C). These findings collectively indicate that ferroptosis plays a significant role in LPS-induced NRCM injury.

Next, to investigate whether ondansetron (OND) attenuates ferroptosis in septic cardiomyopathy, we first established an in vitro model by stimulating NRCMs with LPS. As illustrated in Figure 1D, OND (25 μM or 75 μM) was administered 1 h post-LPS stimulation, and cells were co cultured for 12 h. In comparison to LPS stimulation alone, which increased pro-ferroptotic factors (ACSL4, PTGS2) and decreased antioxidant components (SLC7A11, GPX4), OND treatment partially corrected these changes in a concentration-dependent manner (Figure 1E). Consistent with this, OND treatment attenuated LPS-induced cell death, as reflected by the recovery of Calcein fluorescence and the reduction in PI fluorescence intensity (Figure 1F). Furthermore, LPS stimulation caused a substantial depletion of the antioxidant GSH and an increase in the lipid peroxidation marker MDA, which were normalized by high-concentration (75 μM) but not low-concentration (25 μM) OND treatment (Figure 1G). As expected from the cytoprotective effect, OND treatment at all concentrations tested significantly reduced LPS-induced ROS accumulation in NRCMs, as evidenced by the recovery of DCFH-DA fluorescence (Figure 1H).

In line with this, OND partially reversed the LPS-induced increase in lipid peroxidation, as indicated by the recovery of the Bodipy581/591 C11 red/green fluorescence ratio (Figure 1I). As depicted in Figure 1J, LPS stimulation caused the intracellular accumulation of ferrous iron (Fe^2+^), a characteristic of ferroptosis, as evidenced by the increased FerroOrange fluorescence, which was reduced by OND treatment at all concentrations tested. This was also accompanied by the simultaneous increase in mRNA expression levels of pro-inflammatory cytokines (IL-1α, TNF-α, IL-6, IL-1β) and ferroptosis-related genes (PTGS2, ACSL4), which were partially reversed by OND treatment (Figure 1K). Based on the data above, OND may alleviate ferroptosis in NRCM caused by LPS stimulation and has potential therapeutic effects on septic cardiomyopathy.

### 3.2. Ondansetron Treatment Mitigates Myocardial Ferroptosis in a Mouse Model of Septic Cardiomyopathy (SCM)

**Figure 2:**
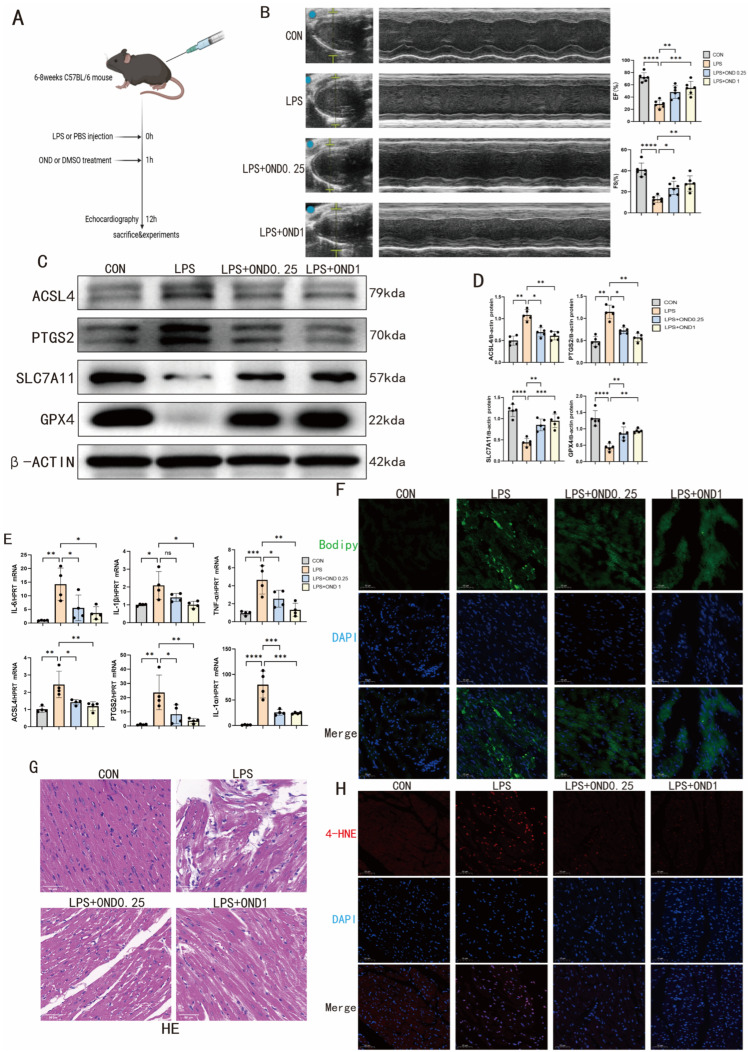
Ondansetron attenuates ferroptosis in cardiomyocytes in vivo in a mouse model of SCM. (**A**) Schematic diagram of the in vivo model of LPS-induced SCM mice. (**B**) Echocardiographic representative images and cardiac function indicators of mice treated with different doses of OND (0.25 mg/kg, 1 mg/kg) or DMSO after intraperitoneal injection of LPS compared with the control group. *N* = 6. Blue dots and yellow marks in B-mode echocardiograms indicate the placement of calipers for anatomical cross-sectional measurements. (**C**,**D**) Representative Western blotting bands and quantitative analysis of ferroptosis markers (ACSL4, PTGS2, SLC7A11, GPX4) in myocardial tissues of mice treated with different doses of OND (0.25 mg/kg, 1 mg/kg) or DMSO after intraperitoneal injection of LPS compared with the control group. *N* = 5. (**E**) The mRNA levels of inflammatory factors and ferroptosis markers after intraperitoneal injection of LPS in the myocardial tissues of mice treated with different doses of OND (0.25 mg/kg, 1 mg/kg) or DMSO, including IL-1α, IL-1β, IL-6, TNF-α, PTGS2, and ACSL4, compared with the control group. *N* = 4. (**F**) Representative images of Bodipy 493/503 staining of mouse hearts treated with different doses of OND (0.25 mg/kg, 1 mg/kg) or DMSO after intraperitoneal injection of LPS, compared with the control group. Scale bar = 50 µm, *N* = 3. Green indicates Bodipy 493/503 (neutral lipids), and blue indicates DAPI (nuclei). (**G**) Representative HE staining graphs of mouse hearts treated with different doses of OND (0.25 mg/kg, 1 mg/kg) or DMSO after intraperitoneal injection of LPS compared with the control group. Scale bar = 50 µm, *N* = 3. (**H**) Representative images of 4-HNE immunofluorescence staining of mouse hearts treated with different doses of OND (0.25 mg/kg, 1 mg/kg) or DMSO after intraperitoneal injection of LPS, compared with the control group. Scale bar = 50 µm, *N* = 3. Red indicates 4-HNE, and blue indicates DAPI (nuclei). For multiple comparisons, one-way or two-way ANOVA was used, followed by Tukey’s multiple comparisons test. A *p*-value of less than 0.05 was considered statistically significant. The data are expressed as the mean ± standard deviation. * *p* < 0.05, ** *p* < 0.01, *** *p* < 0.001, **** *p* < 0.0001, ns, not significant. Data were obtained from independent biological experiments. Black dots represent individual data points.

To further validate the anti-ferroptotic effect of OND in vivo, we also established a murine model of septic cardiomyopathy (SCM) by intraperitoneal injection of LPS. As shown in Figure 2A, OND (0.25 or 1 mg/kg) was given i.p. 1 h after LPS injection, and the heart was collected after 12 h. The LPS-induced impairment of cardiac contractility, reflected by decreased EF% and FS%, was significantly and dose-dependently reversed by OND intervention (Figure 2B). This functional rescue correlated with OND-mediated normalization of key ferroptosis regulators in the heart, including suppression of ACSL4 and PTGS2 overexpression, and restoration of SLC7A11 and GPX4 protein levels (Figure 2C,D). Additionally, the accumulation of iron ions in the hearts of LPS-challenged mice was dose-dependently attenuated by OND, as visualized by Prussian blue staining (Figure S1A).

In mice subjected to LPS challenge, myocardial mRNA levels of pro-inflammatory cytokines (IL-1α, IL-1β, IL-6, TNF-α) and ferroptosis markers (PTGS2, ACSL4) were markedly elevated relative to the controls. OND administration attenuated these transcriptional increases in a dose-dependent fashion (Figure 2E). Parallel observations from Bodipy 493/503 staining revealed that OND, irrespective of concentration, diminished LPS-induced neutral lipid droplet accumulation in cardiac tissue (Figure 2F). Histological examination further corroborated these findings: LPS-triggered myocardial damage—manifesting as disrupted myofiber architecture and intercellular edema—was concurrently ameliorated by OND treatment across all tested doses (Figure 2G). The anti-ferroptotic property of OND in vivo was also confirmed by immunofluorescence staining of 4-HNE, which showed that SCM-induced 4-HNE accumulation in the heart was reduced by OND in a dose-dependent manner (Figure 2H). Collectively, the above results confirm that OND inhibits SCM-induced ferroptosis in the heart.

### 3.3. Oxidative Stress, Mitochondrial Dysfunction, and Endoplasmic Reticulum (ER) Stress Were Significantly Attenuated by Ondansetron in Both NRCM Cells and Mice with SCM

Oxidative stress is a critical mediator of ferroptosis, which is marked by the excessive production of reactive oxygen species (ROS) that drives the ferroptotic reaction [27]. This ROS overproduction leads to damage to mitochondrial architecture and function, and mitochondrial dysfunction, in turn, promotes ROS production [9]. Together, these interconnected events create the necessary conditions for the initiation of ferroptosis [28].

**Figure 3:**
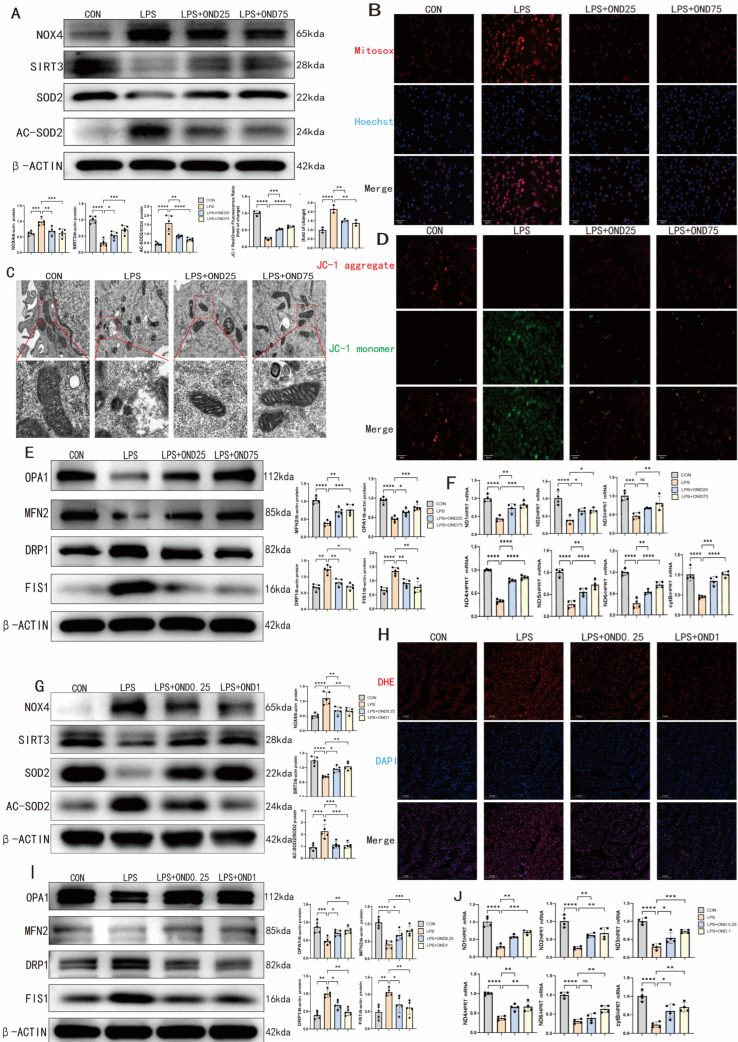
Ondansetron alleviates mitochondrial dysfunction, oxidative stress, and ER stress in both LPS-stimulated NRCMs and the myocardium of mice with SCM. (**A**) Representative Western blotting bands and quantitative analysis of NOX4, SIRT3, Ac-SOD2, and SOD2 after LPS-stimulated NRCM without or with different concentrations of OND (25 μM, 75 μM) treatment. *N* = 5. (**B**) Representative fluorescence images and quantitative analysis of MitoSox treated with or without OND at different concentrations (25 μM, 75 μM) after LPS-stimulated NRCM. Scale bar = 20 µm, *N* = 3. Red indicates MitoSox (mitochondrial superoxide), blue indicates Hoechst (nuclei). (**C**) Representative transmission electron microscope images of NRCM after LPS-stimulated NRCM without or treated with different concentrations of OND (25 μM, 75 μM). Scale bar = 500 nm, *N* = 3. Red lines indicate the contours or boundaries; red frames indicate regions shown at higher magnification in the corresponding insets. (**D**) Representative fluorescence images and quantitative analysis of JC-1 after LPS-stimulated NRCM without or with different concentrations of OND (25 μM, 75 μM). Scale bar = 20 µm, *N* = 3. Red fluorescence indicates JC-1 aggregate (high mitochondrial membrane potential), and green fluorescence indicates JC-1 monomer (low mitochondrial membrane potential). (**E**) Representative Western blotting bands and quantitative analysis of OPA1, MFN2, DRP1, and FIS1 after NRCM stimulated by LPS without or after treatment with different concentrations of OND (25 μM, 75 μM). *N* = 5. (**F**) After LPS-stimulated NRCM, the mRNA levels of MRC complex-related genes (ND1-6, and Cytb) were detected by qRT-PCR either without or with different concentrations of OND (25 μM, 75 μM). *N* = 4. (**G**) Representative Western blot bands and quantitative analyses of NOX4, SIRT3, Ac-SOD2, and SOD2 in the hearts of mice treated with different doses of OND (0.25 mg/kg, 1 mg/kg) or DMSO after intraperitoneal injection of LPS, compared with the control group. *N* = 5. (**H**) Representative fluorescence images of DHE staining of mouse hearts treated with different doses of OND (0.25 mg/kg, 1 mg/kg) or DMSO after intraperitoneal injection of LPS, compared with the control group. Scale bar = 50 µm, *N* = 3. Red indicates DHE (superoxide), blue indicates DAPI (nuclei) (**I**) Representative Western blotting bands and quantitative analysis of OPA1, MFN2, DRP1, and FIS1 in the hearts of mice treated with different doses of OND (0.25 mg/kg, 1 mg/kg) or DMSO after intraperitoneal injection of LPS, compared with the control group. *N* = 5. (**J**) After intraperitoneal injection of LPS, mice were treated with different doses of OND (0.25 mg/kg, 1 mg/kg) or DMSO, and the results were compared to those of the control group. The mRNA levels of MRC complex-related genes (ND1-4, ND6, and Cytb) in myocardial tissue were detected by qRT-PCR. *N* = 4. For multiple comparisons, one-way or two-way ANOVA was used, followed by Tukey’s multiple comparisons test. A *p*-value of less than 0.05 was considered statistically significant. The data are expressed as the mean ± standard deviation. * *p* < 0.05, ** *p* < 0.01, *** *p* < 0.001, **** *p* < 0.0001, ns, not significant. Data were obtained from independent biological experiments. Black dots represent individual data points.

Thus, in the following step, we investigated whether treatment with OND could mitigate oxidative stress and mitochondrial dysfunction in NRCMs and mice with the SCM background. It has been shown that the excessive acetylation of Sirt3 decreases the activity of a major mitochondrial antioxidant enzyme, superoxide dismutase 2 (SOD2) [29]. NOX4 is a member of the NADPH oxidase family and possesses the ability to catalyze the production of an excessive amount of reactive oxygen species (ROS), thereby triggering oxidative stress in cells [30]. LPS stimulation in NRCMs induced a pro-oxidative state characterized by increased NOX4 expression, reduced SIRT3 abundance, and elevated SOD2 acetylation—all of which were partially normalized by OND treatment (Figure 3A). This was accompanied by attenuation of LPS-induced mitochondrial ROS production, as reflected by reduced MitoSOX fluorescence intensity in OND-treated cells (Figure 3B), as well as restoration of Sirt3 mRNA levels (Figure S1B).

Transmission electron microscopy showed that LPS triggered ferroptosis-associated mitochondrial ultrastructural abnormalities in NRCMs—namely shrinkage, membrane densification, and cristae disruption—changes that were mitigated by OND treatment (Figure 3C). This structural preservation was accompanied by functional recovery, as OND partially restored the LPS-induced decline in mitochondrial membrane potential (MMP) reflected by an increased JC-1 red/green fluorescence ratio (Figure 3D). Notably, mitochondrial dynamic imbalance, a recognized component of mitochondrial dysfunction, may underlie these observations [31]. LPS disrupted mitochondrial homeostasis in NRCMs through dual mechanisms: it promoted excessive fission (increased Drp1/Fis1 and decreased MFN2/OPA1 protein levels) and suppressed the transcription of mitochondrial-encoded respiratory chain components (ND1-6, Cytb). OND treatment partially reversed both proteomic alterations (Figure 3E) and transcriptional suppression (Figure 3F). These observations are functionally significant, as downregulation of ND1-6 and Cytb—which encode subunits of MRC complexes I and III—can impair electron transfer and ATP synthesis [32].

Mfn2, a mitochondrial membrane protein involved in ER–mitochondria interactions, is essential for maintaining organellar homeostasis, and Mfn2 deficiency leads to ER stress [33]. Western blot analysis revealed that LPS stimulation significantly upregulated protein expression of the endoplasmic reticulum stress markers ATF6, GRP78, CHOP, and XBP1 in NRCMs. OND treatment partially reversed these elevations, except for ATF6 (Figure S1C). Parallel transcriptomic analysis demonstrated that OND also mitigated the LPS-induced increase in mRNA levels of ATF4, ATF6, GRP78, XBP1 and CHOP (Figure S1D).

The in vivo findings recapitulated our cellular observations. In myocardial tissue from SCM mice, OND treatment normalized the protein expression of NOX4, SIRT3, and acetylated SOD2 (Figure 3G), consistent with its effects in NRCMs. DHE staining further revealed that OND significantly attenuated LPS-induced ROS accumulation in the myocardium (Figure 3H). Parallel analysis of mitochondrial dynamics demonstrated that OND reversed the LPS-triggered imbalance—downregulating fission mediators Drp1 and Fis1 while restoring fusion proteins MFN2 and OPA1 to control levels (Figure 3I). Moreover, OND partially restored the mRNA expression of mitochondrially encoded respiratory chain components (ND1-4, ND6, and Cytb) in SCM hearts (Figure 3J).

In summary, OND alleviates ferroptosis in the setting of septic cardiomyopathy via dual mechanisms: attenuation of oxidative stress and restoration of mitochondrial homeostasis.

### 3.4. RNA-Seq Analysis Identified ATF3 as an Ondansetron-Responsive Differentially Expressed Gene

**Figure 4:**
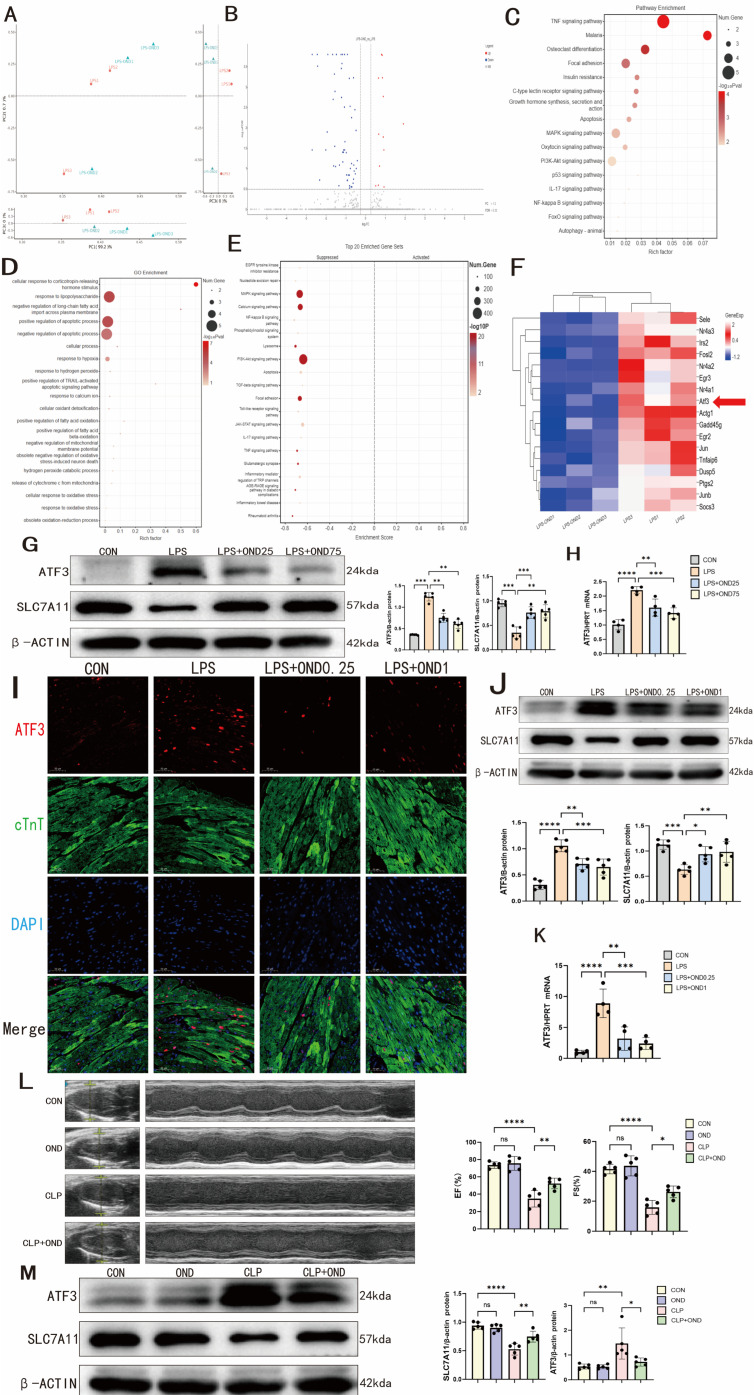
*ATF3* was identified by RNA-seq as a differentially expressed gene after ondansetron treatment, and further experiments confirmed its pivotal role in mitigating ferroptosis. (**A**) PCA categorized the mice into two groups, namely the LPS + DMSO group and the LPS + OND group, with *N* = 3 in each group. (**B**) The differentially expressed genes between the LPS + DMSO and LPS + OND groups are shown in the volcano map. (**C**) Pathway enrichment suggests possible pathway changes in the myocardial tissue of SCM mice after OND treatment. (**D**) GO analysis suggested potential changes in biological processes in the myocardial tissue of SCM mice following OND treatment. (**E**) GSEA enrichment plots showing alterations in gene set profiles in myocardial tissue from SCM mice after OND administration. (**F**) The heat map shows a subset of the differential genes between the LPS + DMSO and LPS + OND groups. (**G**) Representative Western blotting bands and quantitative analysis of ATF3 and SLC7A11 after LPS-stimulated NRCM without or with different concentrations of OND (25 μM, 75 μM). *N* = 5. (**H**) After LPS-stimulated NRCM, the ATF3 mRNA level was detected by qRT-PCR with or without treatment with different concentrations of OND (25 μM, 75 μM). *N* = 4. (**I**) Representative immunofluorescence staining plots of ATF3, cTnT, and DAPI in the hearts of SCM mice treated with different doses of OND (0.25 mg/kg, 1 mg/kg) or DMSO compared with the control group. Scale bar = 50 μM, *N* = 3. Red fluorescence indicates ATF3, green indicates cTnT, and blue indicates DAPI (nuclei) (**J**) Representative Western blotting bands and quantitative analysis of ATF3 and SLC7A11 in the hearts of SCM mice treated with different doses of OND (0.25 mg/kg, 1 mg/kg) or DMSO compared with the control group. *N* = 5. (**K**) Compared with the control group, the hearts of SCM mice were treated with different doses of OND (0.25 mg/kg or 1 mg/kg) or DMSO, and ATF3 mRNA levels were measured by qRT-PCR. *N* = 4. (**L**) Representative echocardiographic images and quantitative analysis of cardiac functional parameters in sham and CLP mice following intraperitoneal injection of OND (1 mg/kg) or DMSO. *N* = 5. Blue dots and yellow marks in B-mode echocardiograms indicate the placement of calipers for anatomical cross-sectional measurements. (**M**) Representative Western blot bands and quantitative analysis of ATF3 and SLC7A11 protein levels in sham and CLP mice treated with intraperitoneal OND (1 mg/kg) or DMSO. *N* = 5. For multiple comparisons, one-way or two-way ANOVA was used, followed by Tukey’s multiple comparisons test. A *p*-value of less than 0.05 was considered statistically significant. The data are expressed as the mean ± standard deviation. * *p* < 0.05, ** *p* < 0.01, *** *p* < 0.001, **** *p* < 0.0001, ns, not significant. Data were obtained from independent biological experiments. Black dots represent individual data points.

To elucidate the transcriptional landscape that contributes to cardioprotection in OND-treated mice, we conducted RNA-seq analysis on cardiac tissues from SCM mice that received either the vehicle (DMSO) or OND. Principal component analysis (PCA) showed a distinct separation between the two groups of treatment, suggesting a difference in their transcriptional expression (Figure 4A). The volcano plot presents a comprehensive list of differentially expressed genes. In the SCM mouse hearts, 57 genes were downregulated, and 13 genes were upregulated following OND treatment (Figure 4B). KEGG pathway analysis revealed that ferroptosis-related pathways were changed after OND treatment, including the TNF, MAPK, P53, and PI3K-AKT signaling pathways (Figure 4C) [34,35,36,37]. GO analysis showed that ferroptosis-related biological processes were significantly enriched, including the cellular response to oxidative stress, negative regulation of mitochondrial membrane potential, and mitochondrial cytochrome c release (Figure 4D) [27,38,39]. GSEA analysis in Figure 4E revealed that after OND treatment, multiple gene sets associated with ferroptosis—including the MAPK signaling pathway, PI3K–AKT signaling pathway, and lysosomes—were significantly suppressed [35,37,40].

Having established that OND mitigates myocardial ferroptosis in murine septic cardiomyopathy through transcriptomic and experimental validation, we next sought to identify its precise molecular targets. RNA-seq analysis revealed that OND treatment significantly downregulated a subset of genes, including activating transcription factor 3 (*ATF3*) (Figure 4F). *ATF3*, a member of the ATF/CREB family, orchestrates diverse cellular processes such as proliferation, apoptosis, and inflammation [41]. Notably, emerging evidence raises the possibility that ATF3 may facilitate ferroptosis through transcriptional downregulation of SLC7A11, an essential component of the cystine/glutamate antiporter (system Xc^−^), thereby potentially weakening cellular antioxidant capacity [42]. Thus, we hypothesized that OND confers anti-ferroptotic effects specifically through downregulation of ATF3.

Validation experiments showed that OND inhibited ATF3 expression in both cellular and mouse models of SCM. In NRCMs, OND abolished the LPS-induced increase in ATF3 expression at both the protein and mRNA levels, and ATF3 expression was inversely proportional to SLC7A11 (Figure 4G,H). In SCM mouse hearts, the ATF3 fluorescence intensity, mainly colocalized with the cardiomyocyte marker cTnT, was increased, but was reduced by OND treatment (Figure 4I). This was corroborated by Western blot and qPCR analyses demonstrating dose-dependent downregulation of ATF3 protein and mRNA in OND-treated myocardial tissue (Figure 4J,K).

To extend our in vitro observations, we employed a cecal ligation and puncture (CLP) model of septic cardiomyopathy in mice. Cardiac function was significantly compromised in CLP mice, reflected by marked reductions in ejection fraction (EF) and fractional shortening (FS); OND treatment conferred partial restoration of these functional parameters (Figure 4L). Furthermore, CLP resulted in elevated ATF3 protein expression and diminished SLC7A11 protein expression in the myocardium, a trend that was partially attenuated by OND intervention (Figure 4M).

These findings indicate that OND alleviates ferroptosis in SCM-induced cardiomyocytes, at least in part, through inhibition of ATF3.

### 3.5. The Protective Effects Conferred by OND on LPS-Challenged NRCMs Were Reversed by ATF3 Overexpression

**Figure 5:**
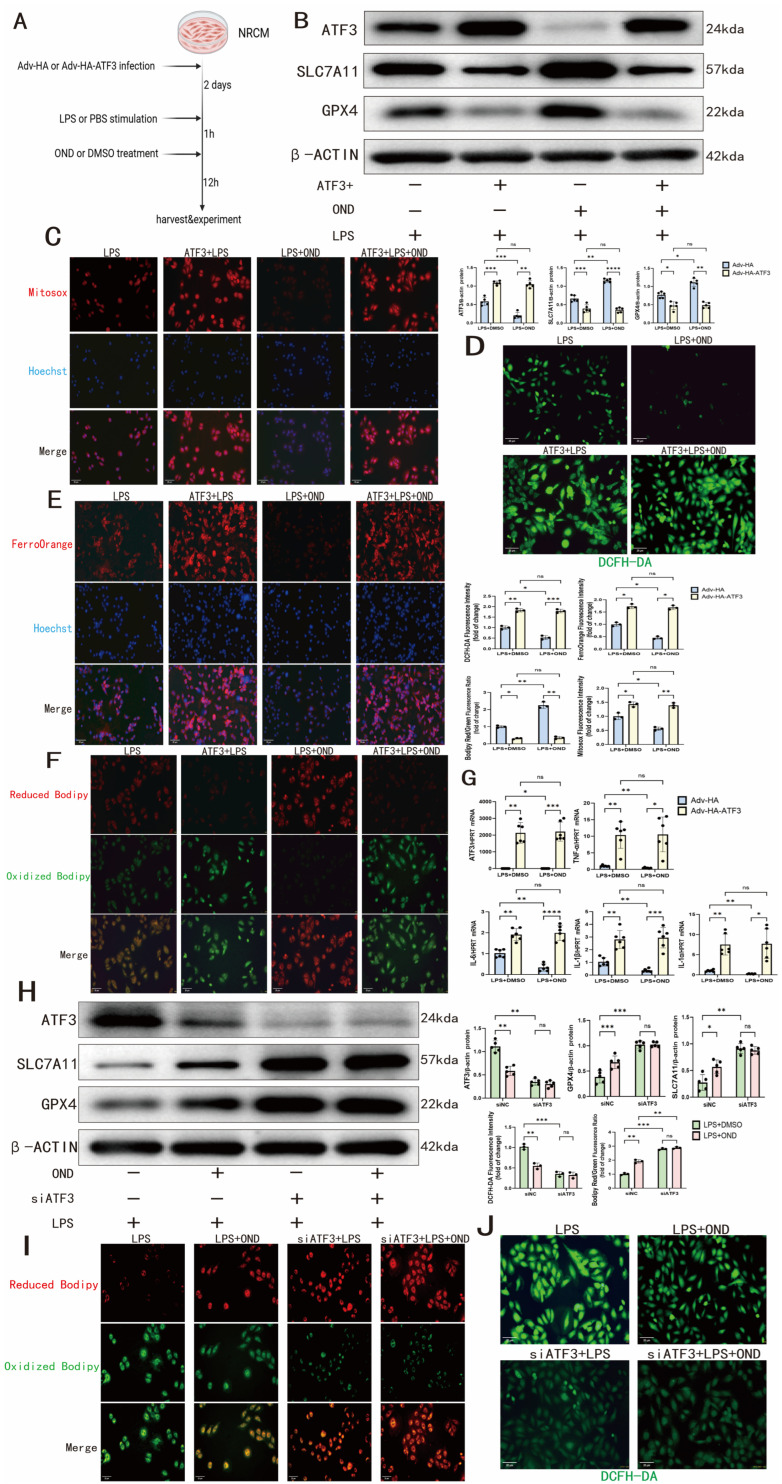
ATF3 overexpression and knockdown of ATF3 by siRNA in NRCMs confirmed its essential role downstream of OND. (**A**) Schematic diagram of NRCMs with overexpression of ATF3 constructed in vitro by adenovirus (Adv). (**B**) Representative Western blotting bands and quantitative analysis of ATF3, SLC7A11, and GPX4 after overexpression of ATF3 and OND treatment (75 μM) in NRCMs under LPS stimulation. *N* = 5. (**C**) Representative fluorescence images and quantitative analysis of MitoSox after overexpression of ATF3 and OND treatment (75 μM) in NRCMs under LPS stimulation. Scale bar = 20 μm, *N* = 3. Red fluorescence indicates MitoSox (mitochondrial superoxide), and blue indicates Hoechst (nuclei). (**D**) Representative fluorescence images and quantitative analysis of DCFH-DA after overexpression of ATF3 and OND treatment (75 μM) in NRCMs under LPS stimulation. Scale bar = 20 μm, *N* = 3. Green fluorescence indicates DCFH-DA (ROS). (**E**) Representative fluorescence images and quantitative analysis of FerroOrange after overexpression of ATF3 and OND treatment (75 μM) in NRCMs under LPS stimulation. Scale bar = 20 μm, *N* = 3. Red fluorescence indicates FerroOrange (Fe^2+^), and blue indicates Hoechst (nuclei). (**F**) Representative fluorescence images and quantitative analysis ofBodipy 581/591 C11 after overexpression of ATF3 and OND treatment (75 μM) in NRCMs under LPS stimulation. Scale bar = 20 μm, *N* = 3. Red fluorescence indicates Reduced Bodipy, and green indicates Oxidized Bodipy (lipid peroxidation). (**G**) Statistical graph of mRNA levels of ATF3, IL-1α, IL-1β, IL-6, and TNF-α detected by qRT-PCR after overexpression of ATF3 and OND treatment (75 μM) in NRCMs under LPS stimulation. *N* = 6. (**H**) Representative Western blot bands and quantitative analysis of ATF3, SLC7A11, and GPX4 in LPS-stimulated NRCMs following siRNA-mediated knockdown of ATF3. *N* = 5. (**I**) Representative DCFH-DA fluorescence images and quantitative analysis in LPS-stimulated NRCMs after ATF3 knockdown by siRNA. Scale bar = 20 μm, *N* = 3. Green fluorescence indicates DCFH-DA (ROS). (**J**) Representative Bodipy581/591 C11 fluorescence images and quantitative analysis in LPS-stimulated NRCMs following siRNA-mediated ATF3 knockdown. Scale bar = 20 μm, *N* = 3. Red fluorescence indicates Reduced Bodipy, and green indicates Oxidized Bodipy (lipid peroxidation). For multiple comparisons, one-way or two-way ANOVA was used, followed by Tukey’s multiple comparisons test. A *p*-value of less than 0.05 was considered statistically significant. The data are expressed as the mean ± standard deviation. * *p* < 0.05, ** *p* < 0.01, *** *p* < 0.001, **** *p* < 0.0001, ns, not significant. Data were obtained from independent biological experiments. Black dots represent individual data points.

By employing an adenoviral strategy to overexpress ATF3 in NRCMs (Adv-HA-ATF3; 48 h before LPS/OND treatment) (Figure 5A), we demonstrated that ATF3 is functionally required for OND-mediated anti-ferroptotic effects. While basal ATF3 overexpression did not alter SLC7A11 or GPX4 protein levels (Figure S2A), it completely reversed OND-induced upregulation of these key ferroptosis inhibitors under LPS stimulation (Figure 5B). Functionally, ATF3 overexpression abolished OND’s ability to suppress mitochondrial and cellular ROS accumulation (MitoSOX, DCFH-DA staining) (Figure 5C,D), ferrous iron accumulation (FerroOrange staining) (Figure 5E), and lipid peroxidation (Bodipy581/591 C11 fluorescence ratio) (Figure 5F). These changes were associated with increased mRNA expression of ATF3 and pro-inflammatory cytokines (IL-1α, IL-1β, IL-6, and TNF-α) regardless of OND treatment (Figure 5G).

To validate the essential function of ATF3, siRNA-mediated knockdown of ATF3 was performed in NRCMs. As illustrated in Figure 5H, depletion of ATF3 led to pronounced upregulation of SLC7A11 and GPX4 protein levels following LPS challenge. In ATF3-silenced cells, subsequent OND treatment failed to further reduce ATF3 expression or enhance the protective effect against ferroptosis. Oxidative stress and lipid peroxidation, assessed by DCFH-DA and Bodipy 581/591 C11 staining respectively, were significantly alleviated by siATF3 in LPS-treated NRCMs (Figure 5I,J). Under these conditions, OND failed to confer any additional protective benefit.

Taken together, these results demonstrate that ATF3 is essential for the ferroptosis-attenuating effect of OND in an in vitro model of LPS-challenged NRCMs.

### 3.6. Cardiomyocyte-Specific ATF3 Overexpression in SCM Mice Not Only Negated the Therapeutic Effect of OND but Also Aggravated Cardiac Ferroptosis

**Figure 6:**
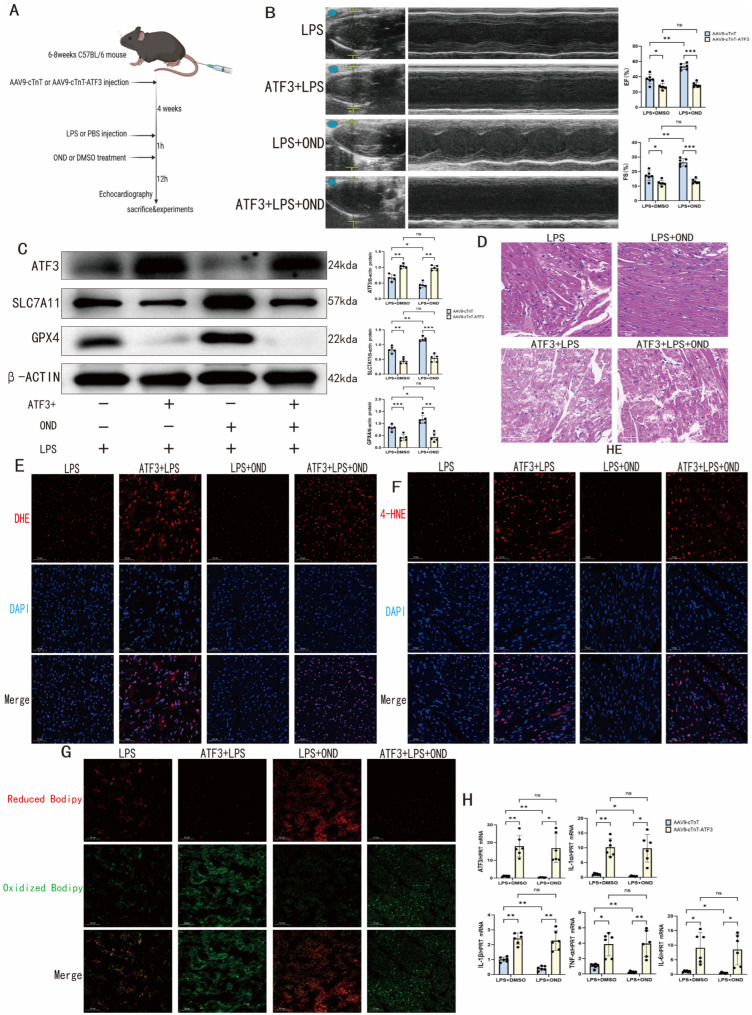
The specific overexpression of ATF3 in cardiomyocytes counteracted the therapeutic effects of ondansetron and further aggravated cardiac ferroptosis in a murine model of SCM. (**A**) Schematic diagram of overexpression of ATF3 in SCM mice via adeno-associated virus 9 (AAV9). (**B**) In SCM mice, after cardiomyocyte-specific overexpression of ATF3 and OND treatment (1 mg/kg), representative echocardiographic images and cardiac function indicators were obtained. *N* = 6. Blue dots and yellow marks in B-mode echocardiograms indicate the placement of calipers for anatomical cross-sectional measurements. (**C**) Representative Western blotting bands and quantitative analysis of ATF3, SLC7A11, and GPX4 after cardiomyocyte-specific overexpression of ATF3 and treatment with OND (1 mg/kg) in SCM mice. *N* = 5. (**D**) Representative images of HE staining in SCM mice after cardiomyocyte-specific overexpression of ATF3 and treatment with OND (1 mg/kg). Scale bar = 50 μM, *N* = 3. (**E**) Representative images of DHE staining in SCM mice after cardiomyocyte-specific overexpression of ATF3 and treatment with OND (1 mg/kg). Scale bar = 50 μm, *N* = 3. Red indicates DHE (superoxide), blue indicates DAPI (nuclei). (**F**) Representative images of 4-HNE immunofluorescence staining in SCM mice after cardiomyocyte-specific overexpression of ATF3 and treatment with OND (1 mg/kg). Scale bar = 50 μm, *N* = 3. Red fluorescence indicates 4-HNE, and blue indicates DAPI (nuclei). (**G**) Representative images of Bodipy581/591 C11 staining in SCM mice after cardiomyocyte-specific overexpression of ATF3 and treatment with OND (1 mg/kg). Scale bar = 50 μm, *N* = 3. Red fluorescence indicates Reduced Bodipy, and green indicates Oxidized Bodipy (lipid peroxidation). (**H**) In SCM mice, after cardiomyocyte-specific overexpression of ATF3 and treatment with OND (1 mg/kg), qRT-PCR was used to detect mRNA levels of ATF3, IL-1α, IL-1β, IL-6, and TNF-α. *N* = 6. For multiple comparisons, one-way or two-way ANOVA was used, followed by Tukey’s multiple comparisons test. A *p*-value of less than 0.05 was considered statistically significant. The data are expressed as the mean ± standard deviation. * *p* < 0.05, ** *p* < 0.01, *** *p* < 0.001, ns, not significant. Data were obtained from independent biological experiments. Black dots represent individual data points.

To conclusively establish ATF3 as a critical mediator of OND’s therapeutic action in vivo, we generated an adeno-associated virus (AAV9) encoding ATF3 under the control of the cardiac-specific troponin T (cTnT) promoter. Mice received tail vein injections of AAV9-cTnT-ATF3 or control virus, followed by a 4-week transduction period prior to SCM induction and OND treatment (Figure 6A). Under physiological conditions, cardiac-specific ATF3 overexpression alone did not significantly alter cardiac function (Figure S2B). However, in the setting of SCM, ATF3-overexpressing mice exhibited exacerbated cardiac dysfunction—evidenced by further reductions in EF% and FS%—that was refractory to OND treatment (Figure 6B). This functional deterioration correlated with complete abrogation of OND’s anti-ferroptotic effects, as ATF3 overexpression suppressed SLC7A11 and GPX4 protein levels despite OND administration (Figure 6C). HE staining showed that cardiac-specific ATF3 overexpression exacerbated myocardial injury in SCM mice, with severe architectural abnormalities, including profound myofiber disarray and increased interstitial edema (Figure 6D). Mechanistically, ATF3 overexpression potentiated oxidative stress (increased DHE fluorescence) (Figure 6E) and lipid peroxidation (increased 4-HNE, decreased Bodipyred/green ratio) (Figure 6F,G), independently of OND treatment. Transcript analysis also confirmed the induction of pro-inflammatory cytokines (IL-1α, IL-1β, IL-6, TNF-α) in ATF3-overexpressing myocardium (Figure 6H). Collectively, these in vivo findings validate ATF3 as a pivotal node in the OND-targeted pathway, demonstrating that cardiomyocyte-specific ATF3 overexpression both exacerbates ferroptotic injury and completely abolishes the therapeutic effects of OND.

### 3.7. By Targeting and Antagonizing HTR3A, Ondansetron Confers Protection Against Ferroptosis

**Figure 7:**
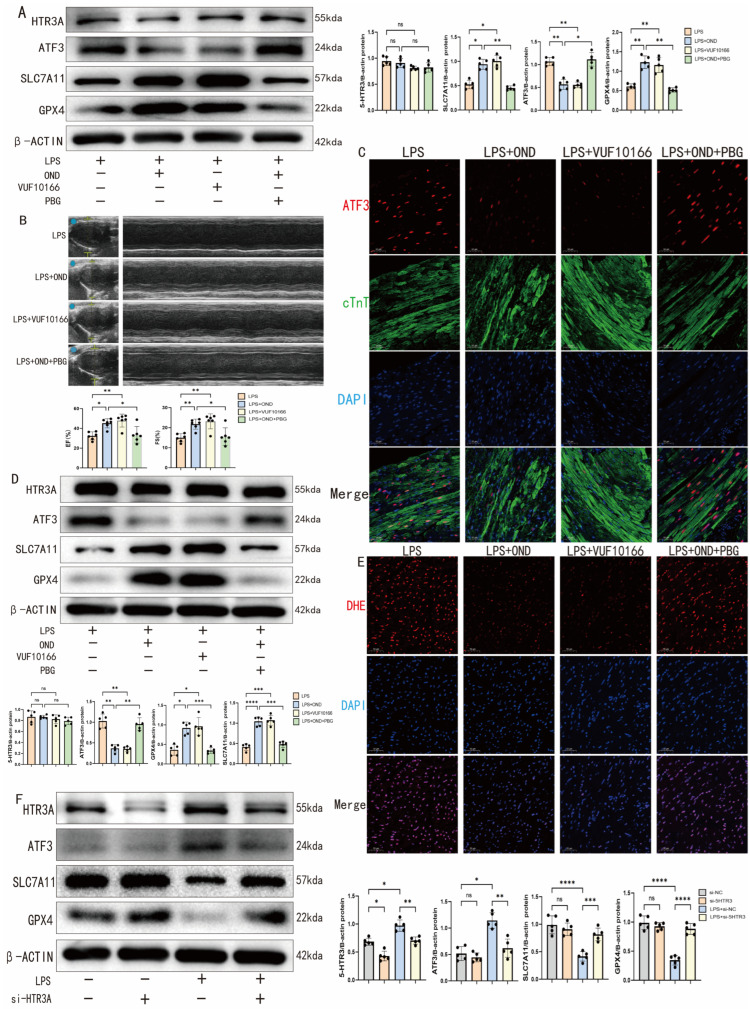
The anti-ferroptotic effect of ondansetron is mediated through HTR3A antagonism. (**A**) Representative Western blotting bands and quantitative analysis of HTR3A, ATF3, SLC7A11, and GPX4 after OND treatment (75 μM), VUF 10166 treatment (0.1 μM), or PBG treatment (50 μM) in NRCMs under LPS stimulation. *N* = 5. (**B**) Representative echocardiographic images and cardiac function indicators of SCM mice after OND treatment (1 mg/kg), VUF 10166 treatment (0.1 mg/kg), or PBG treatment (1 mg/kg). *N* = 6. Blue dots and yellow marks in B-mode echocardiograms indicate the placement of calipers for anatomical cross-sectional measurements. (**C**) Representative immunofluorescence staining patterns of ATF3, cTnT, and DAPI in SCM mice after treatment with OND (1 mg/kg), VUF 10166 (0.1 mg/kg), or PBG (1 mg/kg). Scale bar = 50 μm, *N* = 3. Red fluorescence indicates ATF3, green indicates cTnT, and blue indicates DAPI (nuclei). (**D**) Representative Western blotting bands and quantitative analysis of HTR3A, ATF3, SLC7A11, and GPX4 in SCM mice after treatment with OND (1 mg/kg), VUF 10166 (0.1 mg/kg), or PBG (1 mg/kg). *N* = 5. (**E**) Representative DHE staining patterns of SCM mice after OND treatment (1 mg/kg), VUF 10166 treatment (0.1 mg/kg), or PBG treatment (1 mg/kg). Scale bar = 50 μm, *N* = 3. Red indicates DHE (superoxide), blue indicates DAPI (nuclei). (**F**) Representative Western blotting bands and quantitative analysis of HTR3A, ATF3, SLC7A11, and GPX4 in NRCMs under LPS stimulation and treatment with siRNA targeting HTR3A. *N* = 5. For multiple comparisons, one-way or two-way ANOVA was used, followed by Tukey’s multiple comparisons test. A *p*-value of less than 0.05 was considered statistically significant. The data are expressed as the mean ± standard deviation. * *p* < 0.05, ** *p* < 0.01, *** *p* < 0.001, **** *p* < 0.0001, ns, not significant. Data were obtained from independent biological experiments. Black dots represent individual data points.

While ondansetron is classically recognized as a selective 5 HT3 receptor antagonist at clinically relevant doses, it exhibits broader receptor interactions at higher concentrations, including antagonism of the rapid delayed rectifier potassium channel (hERG) on cardiomyocyte membranes as well as GABA and glycine receptors [15,43]. To determine whether ondansetron inhibits ATF3 by antagonizing the 5-HT3 receptor, we employed VUF 10166, a novel HTR3A (the core subunit of the 5-HT3 receptor) antagonist, and 1-phenylbiguanide (PBG), the 5-HT3 receptor agonist, for validation. In vitro, VUF 10166 mimicked OND’s effects in LPS challenged NRCMs—downregulating ATF3 and upregulating SLC7A11/GPX4—while PBG co treatment reversed OND mediated protection (Figure 7A). In vivo, VUF 10166 recapitulated OND’s therapeutic benefits in SCM mice, including preserved cardiac function (Figure 7B), reduced myocardial ATF3 expression (Figure 7C,D), and restored SLC7A11/GPX4 levels (Figure 7D). DHE staining also indicated that both OND and VUF 10166 treatments alleviated oxidative stress in the myocardial tissue of SCM mice (Figure 7E). Consistent with these observations, PBG co administration abolished OND’s protective effects across all measured parameters (Figure 7B–E). To further interrogate the functional role of HTR3A in OND-mediated cardioprotection, we employed a genetic silencing approach using small interfering RNA (siRNA) targeting HTR3A in NRCMs. Transfection with HTR3A-specific siRNA effectively reduced HTR3A protein expression, which was otherwise upregulated upon LPS stimulation. While HTR3A knockdown exerted minimal effects under basal conditions, it significantly attenuated LPS-induced ATF3 upregulation and consequently restored SLC7A11 and GPX4 expression (Figure 7F). In summary, these findings establish that OND mitigates ferroptosis through HTR3A antagonism and subsequent ATF3 downregulation, identifying the *HTR3A/ATF3* axis as a critical pathogenic pathway in septic cardiomyopathy.

## 4. Discussion

Our findings suggest that ondansetron (OND) may exert beneficial effects in a murine model of septic cardiomyopathy (SCM) by suppressing cardiomyocyte ferroptosis. The observed cytoprotection appears to involve antagonism of the HTR3A receptor and subsequent downregulation of ATF3, which may attenuate ferroptotic cell death through two potential complementary mechanisms: the first is restoration of GPX4-mediated antioxidant defense via upregulation of SLC7A11/system Xc^−^ [44], and the other is amelioration of mitochondrial dysfunction to limit ROS production and oxidative stress (Graphical Abstract). Our work not only expands the potential application of this classic 5-HT3 receptor antagonist to cardiac therapy, emphasizing its efficacy in SCM treatment, but also reveals the *HTR3A/ATF3* pathway as a novel mechanism contributing to ferroptosis in cardiomyocytes, offering a potential target for intervention.

Ferroptosis, an iron-dependent, lipid peroxidation-driven form of programmed cell death, has rapidly emerged as a research focus in cardiac disease [6]. Stress-mediated disruption of the GPX4/GSH pathway, as well as metabolic and iron disturbances, contribute to ferroptosis in heart disease by facilitating lipid peroxidation through the Fenton reaction and subsequent membrane rupture [44]. Ferroptosis research has great potential in the cardiovascular community, particularly in sepsis-induced cardiomyopathy, and its inhibition is essential for heart disease therapy [21]. In this study, we found that OND can protect mice from sepsis-induced cardiomyopathy by inhibiting ferroptosis in cardiomyocytes.

A central feature of septic cardiomyopathy is the establishment of a vicious cycle wherein mitochondrial dysfunction, oxidative stress, and inflammation perpetuate one another. Mitochondrial damage, manifested by impaired dynamics, depolarization, and electron transport chain failure, fuels ROS overproduction and iron dysregulation [4,45]. This oxidative environment not only directly compromises contractile function but also creates the biochemical conditions necessary for ferroptosis—specifically, GSH depletion, GPX4 inactivation, and accumulation of lipid peroxides [9,46]. Ferroptosis, in turn, exacerbates mitochondrial injury through lipid peroxide-mediated membrane damage, thereby closing a positive feedback loop [27,44]. Inflammation further amplifies this cycle by suppressing mitochondrial biogenesis and enhancing ROS generation [47,48]. Our study demonstrates that OND interrupts this self-amplifying loop at multiple levels: it restores mitochondrial integrity, reduces both cytosolic and lipid ROS, and reinforces SLC7A11/GPX4 antioxidant defense via ATF3 suppression. This suggests that ferroptosis inhibition is the predominant mechanism of OND-mediated cardioprotection, with anti-inflammatory and general antioxidant effects representing ancillary benefits. Consequently, in its attempt to address the intersection between mitochondrial function and ferroptosis, OND offers a comprehensive approach to treating septic cardiomyopathy. In SCM, ER stress is a secondary effect of mitochondrial dysfunction and oxidative stress, which is mediated by the loss of MFN2-dependent mitochondria-ER coupling and direct protein misfolding induced by ROS, which collectively overwhelm ER proteostasis [49,50,51]. In our study, we also observed that OND treatment modestly alleviated ER stress in cardiomyocytes. However, due to the limited effect, we believe that this may be an indirect effect due to the improvement of mitochondrial function and oxidative stress by OND.

ATF3, a transcription factor belonging to the ATF/CREB family, is an “early response gene” induced by various forms of cellular stresses, such as oxidative stress, mitochondrial dysfunction, and endoplasmic reticulum stress [41,52,53]. Recent research has shown that ATF3 is a critical regulator that promotes ferroptosis by inhibiting System Xc^−^ [42,54,55]. Our work demonstrates that OND may protect cardiomyocytes from ferroptosis in septic cardiomyopathy by suppressing ATF3, thereby recovering mitochondrial function and reducing oxidative stress.

As the sole ionotropic serotonin receptor (Cys-loop receptor family), the pentameric 5-HT_3_ ligand-gated channel transmits fast-excitatory signals [56]. The critical HTR3A subunit determines the cation channel framework, and the regulatory HTR3B subunit modulates its functional characteristics and cellular localization [57]. Ondansetron (OND), a classic 5-HT_3_ receptor antagonist, has been demonstrated to exert anti-inflammatory effects in recent research and has been validated in animal models of both acute pancreatitis and benign prostatic hyperplasia [13,58]. However, the application of ondansetron in the cardiovascular field remains restricted. Our study revealed that OND ameliorates septic cardiomyopathy (SCM) by inhibiting cardiomyocyte ferroptosis via the HTR3A/ATF3 pathway, thus mitigating mitochondrial dysfunction and alleviating oxidative stress.

Our findings in this study suggest that ondansetron may exhibit a multifaceted profile, encompassing anti-inflammatory, anti-oxidative, and anti-ferroptotic properties. Given that inflammation, oxidative stress, and ferroptosis are closely interwoven in septic cardiomyopathy, we employed a variety of complementary assays and end-points to probe the existence of each effect. With regard to inflammation, OND treatment was accompanied by decreased mRNA expression of the LPS-induced pro-inflammatory cytokines IL-1β, IL-6, and TNF-α. Concerning oxidative stress, we observed diminished DCFH-DA and MitoSOX fluorescence signals in OND-treated cells, along with altered expression of proteins implicated in redox regulation, including NOX4, SIRT3, and SOD2. In terms of ferroptosis, we detected changes in key ferroptosis-associated proteins (ACSL4, PTGS2, GPX4, and SLC7A11), as well as reductions in intracellular free Fe^2+^ (FerroOrange), lipid peroxidation (Bodipy581/591 C11), and 4-HNE levels. Taken together, these observations raise the interesting possibility that ondansetron may exert a previously unrecognized anti-ferroptotic effect in cardiomyocytes. In our in vitro experiments, NRCMs were treated with OND at concentrations of 25 μM and 75 μM, selected based on the manufacturer’s information and the previous literature [59]. These concentrations were confirmed to have no significant cytotoxicity by the CCK-8 assay (Figure S3). Our results demonstrate that 25 μM OND is sufficient to elicit an anti-ferroptosis effect in NRCMs, while 75 μM produces a more pronounced and stable protective outcome. We administered OND at 0.25 and 1 mg/kg in vivo, doses referenced to clinical practice and prior studies [59,60]. Our results indicate that treatment with the lower concentration (0.25 mg/kg) is sufficient to confer an anti-ferroptosis benefit in the SCM mouse model.

Although ondansetron exhibits high selectivity for the 5-HT_3_ receptor (IC_50_ ≈ 6.4 nM) [60], off-target effects at elevated concentrations have been documented in the literature. These include inhibition of adult and fetal muscle-type nicotinic acetylcholine receptors (nAChR; IC_50_ = 14.2 μM and 16.0 μM, respectively) and blockade of voltage-gated sodium channels (neural IC_50_ = 12 μM; cardiac IC_50_ = 88.5 μM) [43,61,62]. To exclude off-target interference, we complemented our approach with VUF10166 and phenylbiguanide (PBG). VUF10166 is a novel, high-affinity 5-HT_3_ receptor ligand (K_i_ = 0.04 nM at 5-HT_3_A, 22 nM at 5-HT_3_AB), whereas PBG is a canonical selective 5-HT_3_ agonist (EC_50_ ≈ 3.0 μM at human 5-HT_3_A) [16,63]. Both compounds are extensively utilized in biological assays to antagonize or activate 5-HT_3_ receptors, respectively [17,64,65,66]. In our study, in LPS-challenged neonatal rat cardiomyocytes (NRCM), VUF10166 recapitulated the cardioprotective phenotype of ondansetron, while sequential application of PBG partially abrogated this benefit. These results strongly suggest that the anti-ferroptotic activity of ondansetron is conferred by on-target 5-HT_3_ receptor antagonism, independent of off-target effects.

While there has been no experimental elucidation of the mechanistic connection between the antagonism of the HTR3A gene and the inhibition of ATF3 expression, a plausible signaling mechanism can be inferred from published evidence. The 5-HT_3_ receptor is a Ca^2+^-permeable ligand-gated cation channel whose activation promotes Ca^2+^ influx via the receptor pore, L-type calcium channels, and ryanodine receptor-mediated Ca^2+^-induced Ca^2+^ release [67]. This amplifies downstream Ca^2+^-dependent signaling, including CaMKIIα and ERK1/2 phosphorylation—responses that are blocked by 5-HT_3_ antagonists [68]. In parallel, the link between calcium signaling and ATF3 transcription has been well delineated in the nervous system, where ATF3 is a direct transcriptional target of the cAMP response element-binding protein (CREB) [69]. Calcium influx generates nuclear calcium transients that activate Ca^2+^/calmodulin-dependent protein kinase IV (CaMKIV), which phosphorylates CREB and promotes its binding to the canonical cyclic AMP response element (CRE) within the ATF3 proximal promoter [70]. Therefore, we speculate that LPS stimulation of NRCMs may trigger 5-HT_3_ receptor-dependent Ca^2+^ entry, activating CaMKII/ERK and the nuclear CaMKIV-CREB axis to induce ATF3 transcription. It should be noted that ATF3 expression is also subject to regulation by other stress-responsive pathways, including JNK and p38 MAPK cascades, which are likewise activated upon LPS challenge [71,72]. Whether 5-HT_3_ receptor-mediated signals converge with these parallel pathways, and whether ondansetron exerts its anti-ferroptotic actions via such cross-talk, remains an open question requiring further investigation.

A potential limitation of this study is the species discrepancy between in vitro NRCMs (neonatal rat) and in vivo septic cardiomyopathy models (adult C57BL/6 mouse). However, the *HTR3A/ATF3* axis is likely conserved across these rodents: HTR3A shares >90% amino acid identity between rats and mice, with identical ligand-binding domains, and ondansetron exhibits similar pharmacology in both species [73,74]. ATF3 is an evolutionarily conserved stress-responsive transcription factor that is rapidly induced by LPS and cytokines in rat, mouse, and human cells [75]. Both rat and mouse cardiomyocytes upregulate ATF3 under pathological stress, and ATF3 regulates cell survival pathways across species [76]. Nonetheless, subtle species-dependent variations in ATF3-governed transcriptional programs cannot be definitively excluded, nor can potential differences in 5-HT_3_ receptor expression or coupling efficiency between neonatal and adult cardiomyocytes.

Our study has some limitations. First, the exact pathway by which HTR3A antagonism reduces ATF3 expression remains to be elucidated. Additionally, the majority of our in vitro and in vivo experiments utilized an LPS-induced endotoxemic septic cardiomyopathy model. Although we have partially validated the principal findings in the more clinically relevant CLP sepsis model, comprehensive corroboration in this system represents an important avenue for future work. These points will be the focus of future investigations. Second, all in vitro experiments were performed using neonatal rat cardiomyocytes (NRCMs). Compared with adult cardiomyocytes, NRCMs exhibit distinct metabolic profiles, lower mitochondrial density, and immature calcium handling, which may influence susceptibility to ferroptosis and the observed cardioprotective effects [77]. Validation in adult cardiomyocytes or hiPSC-CMs is therefore warranted. Third, HTR3A involvement is supported by pharmacological and siRNA-based approaches rather than genetic ablation. While multiple 5-HT_3_ receptor modulators and scrambled siRNA controls reinforce target specificity, definitive validation requires cardiac-specific HTR3A knockout studies, which were beyond the scope of the present work.

## 5. Conclusions

Taken together, our research proves that the treatment of OND has a positive effect on SCM injury by reducing cardiomyocyte ferroptosis. The mechanism of action involves the antagonism of the HTR3A receptor by OND, leading to suppressed ATF3 expression, which in turn elicits a cascade of beneficial downstream effects, including the normalization of mitochondrial function, a reduction in oxidative stress, and an improvement in endoplasmic reticulum stress. These collective effects ultimately rescue cells from ferroptosis. The significance of our work lies in its dual contribution: it pioneers the association between OND and the key regulator ATF3, which may expand the application of OND, and introduces a new treatment strategy for SCM, thus paving the way for future clinical studies.

## Data Availability

The original contributions presented in this study are included in the article/Supplementary Materials. Further inquiries can be directed to the corresponding authors.

## Supplementary Materials

The following supporting information can be downloaded at: https://www.mdpi.com/article/10.3390/biomedicines14051040/s1, Figure S1: Ondansetron attenuates LPS induced iron deposition and ER stress in cardiomyocytes. (A) Representative Prussian blue staining of mouse hearts treated with different doses of OND (0.25 mg/kg, 1 mg/kg) or DMSO after intraperitoneal injection of LPS compared with the control group. Scale bar = 50 µm, *N* = 3. (B) After LPS-stimulated NRCM, SIRT3 mRNA levels were detected by qRT-PCR with or without treatment with different concentrations of OND (25 µM, 75 µM). *N* = 4. (C) Representative Western blotting bands and quantitative analysis of endoplasmic reticulum stress-related proteins ATF6, GRP78, CHOP, and XBP1 after LPS-stimulated NRCM without or after treatment with different concentrations of OND (25 µM, 75 µM). *N* = 4. (D) After LPS-stimulated NRCM, the mRNA levels of ATF4, ATF6, GRP78, CHOP, and XBP1 were detected by qRT-PCR without or after treatment with different concentrations of OND (25 µM, 75 µM). *N* = 4. For multiple comparisons, one-way or two-way ANOVA was used, followed by Tukey’s multiple comparisons test. A *p*-value of less than 0.05 was considered statistically significant. The data are expressed as the mean ± standard deviation. * *p* < 0.05, ** *p* < 0.01, *** *p* < 0.001, **** *p* < 0.0001. Data were obtained from independent biological experiments; Figure S2: Specific overexpression of ATF3 in mouse cardiomyocytes under basal physiological conditions. (A) Representative western blotting bands and quantitative analysis of ATF3, SLC7A11, and GPX4 after overexpression of ATF3 and OND treatment (75 µM) in NRCMs under physiological conditions. *N* = 4. (B) Under physiological conditions, after cardiomyocyte-specific ATF3 overexpression, representative echocardiographic images and cardiac function indicators were obtained. *N* = 5. For multiple comparisons, one-way or two-way ANOVA was used, followed by Tukey’s multiple comparisons test. A *p*-value of less than 0.05 was considered statistically significant. The data are expressed as the mean ± standard deviation. * *p* < 0.05, ** *p* < 0.01, *** *p* < 0.001, **** *p* < 0.0001. Data were obtained from independent biological experiments. Figure S3: Effect of OND on the viability of NRCMs. NRCMs were treated with various concentrations of OND for 24 h. Subsequently, cell viability was assessed using the CCK-8 assay kit (C0037, Beyotime, Shanghai, China) according to the manufacturer’s instructions. Table S1: Antibodies for Western blotting. Tables S2 and S3: Primers for Real-Time PCR. Table S4: Antibodies for Immunofluorescence.

## Abbreviations

The following abbreviations are used in this manuscript:

SCM	Septic Cardiomyopathy
GPX4	Glutathione peroxidase 4
GSH	Glutathione
CLP	Cecal Ligation and Puncture
ROS	Reactive Oxygen Species
DAMPs	Damage-Associated Molecular Patterns
NF-κB	Nuclear Factor Kappa-Light-Chain-Enhancer of Activated B Cells
NLRP3	NLR Family Pyrin Domain-Containing 3
OND	Ondansetron
5-HT3	5-Hydroxytryptamine 3
HTR3A	5-Hydroxytryptamine Receptor 3A
ATF3	Activating Transcription Factor 3
SLC7A11	Solute Carrier Family 7 Member 11
PTGS2	Prostaglandin-Endoperoxide Synthase 2
ACSL4	Acyl-CoA Synthetase Long-Chain Family Member 4
OPA1	Optic Atrophy 1
MFN2	Mitofusin 2
DRP1	Dynamin-Related Protein 1
FIS1	Mitochondrial Fission 1 Protein
NOX4	NADPH Oxidase 4
SOD2/AC-SOD2	Superoxide Dismutase 2/Acetylated SOD2
SIRT3	Sirtuin 3
ND1-6	NADH Dehydrogenase (Ubiquinone) Subunits 1-6
CytB	Cytochrome b
ATF4	Activating Transcription Factor 4
ATF6	Activating Transcription Factor 6
GRP78	Glucose-Regulated Protein 78
CHOP	C/EBP Homologous Protein
XBP1	X-Box Binding Protein 1
MMP	Mitochondrial Membrane Potential
ATP	Adenosine Triphosphate
Con	Control
LPS	Lipopolysaccharide
NRCMs	Neonatal Rat Cardiomyocytes
MDA	Malondialdehyde
EF	Ejection Fraction
FS	Fractional Shortening
4-HNE	4-hydroxy-2-nonenal
cTnT	Cardiac Troponin T
ER	Endoplasmic Reticulum
IL-1α, IL-1β, IL-6	Interleukin 1 Alpha, 1- Beta and 6
TNF-α	Tumor Necrosis Factor Alpha
PCA	Principal Component Analysis
GO	Gene Ontology
PBG	1-phenylbiguanide
ADV	Adenovirus
AAV9	Adeno-Associated Virus Serotype 9
HPRT	Hypoxanthine–Guanine Phosphoribosyltransferase
