# Supplementary material for: Ferroptosis in Septic Cardiomyopathy Is Alleviated by Ondansetron: The Critical Role of the HTR3A-ATF3 Axis in Mitochondrial and Oxidative Homeostasis
Source: Biomedicines. 2026 May 3;14(5):1040. doi: 10.3390/biomedicines14051040 (PMC13204977; doi:10.3390/biomedicines14051040)
Supplement: Supplementary file 1 [file biomedicines-14-01040-s001.zip › biomedicines-4203716-supplementary.pdf]

Figure S1

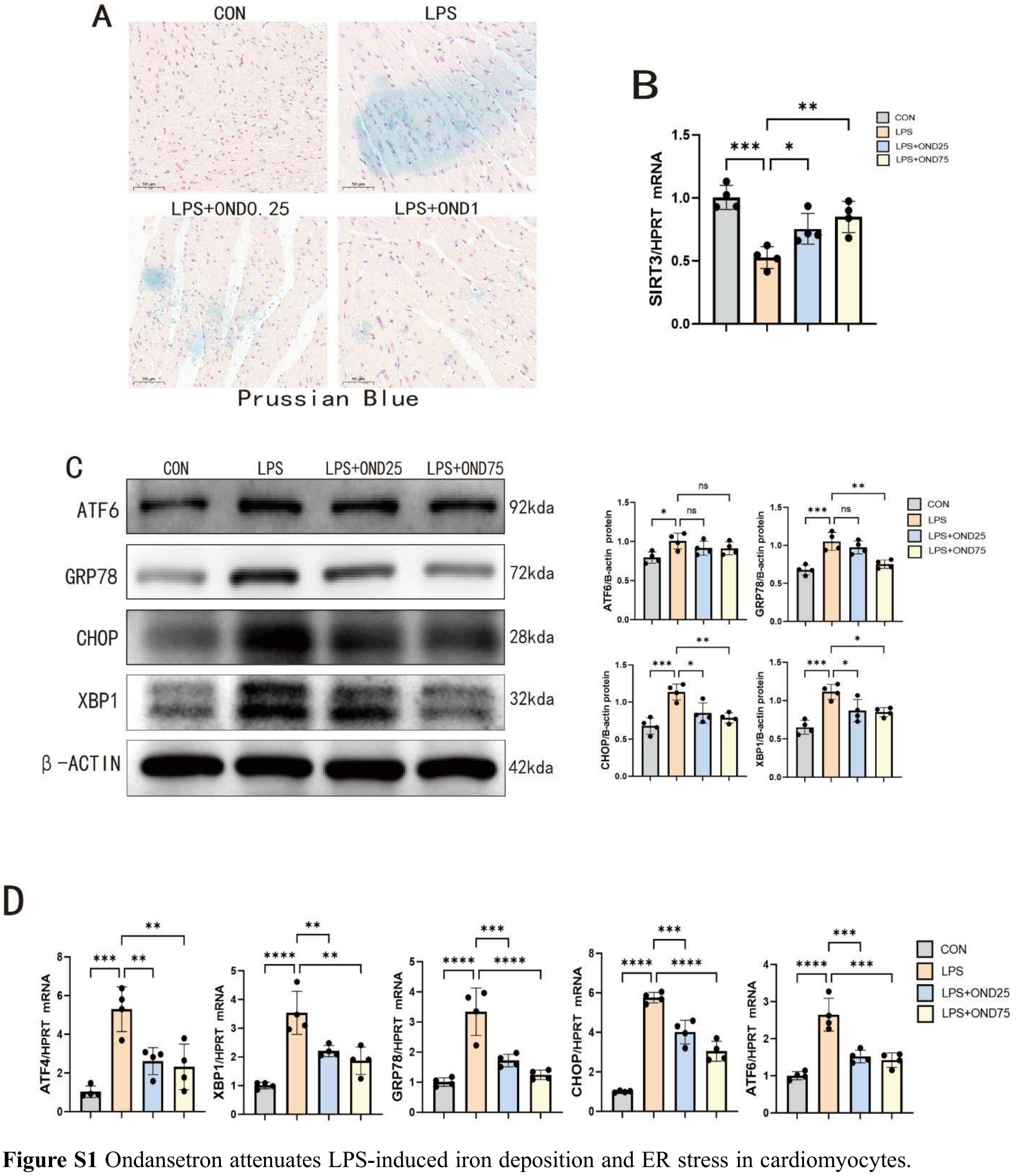

Figure S1 Ondansetron attenuates LPS-induced iron deposition and ER stress in cardiomyocytes. (A) Representative Prussian blue staining of mouse hearts treated with different doses of OND (0.25mg/kg, 1mg/kg) or DMSO after intraperitoneal injection of LPS compared with the control group. Scale bar = 50 um, N=3. (B) After LPS-stimulated NRCM, SIRT3 mRNA levels were detected by qRT-PCR with or without treatment with different concentrations of OND (25uM,

75uM). N=4. (C) Representative Western blotting bands and quantitative analysis of endoplasmic reticulum stress-related proteins ATF6, GRP78, CHOP, and XBP1 after LPS-stimulated NRCM without or after treatment with different concentrations of OND (25uM,75uM). N=4. (D) After LPS-stimulated NRCM, the mRNA levels of ATF4, ATF6, GRP78, CHOP, and XBP1 were detected by qRT-PCR without or after treatment with different concentrations of OND (25uM, 75uM). N=4. For multiple comparisons, one-way or two-way ANOVA was used, followed by Tukey's multiple comparisons test. A p-value of less than 0.05 was considered statistically significant. The data are expressed as the mean ± standard deviation. * $P<0.05$, ** $P<0.01$, *** $P<0.001$, **** $P<0.0001$. Data were obtained from independent biological experiments.

Figure S2

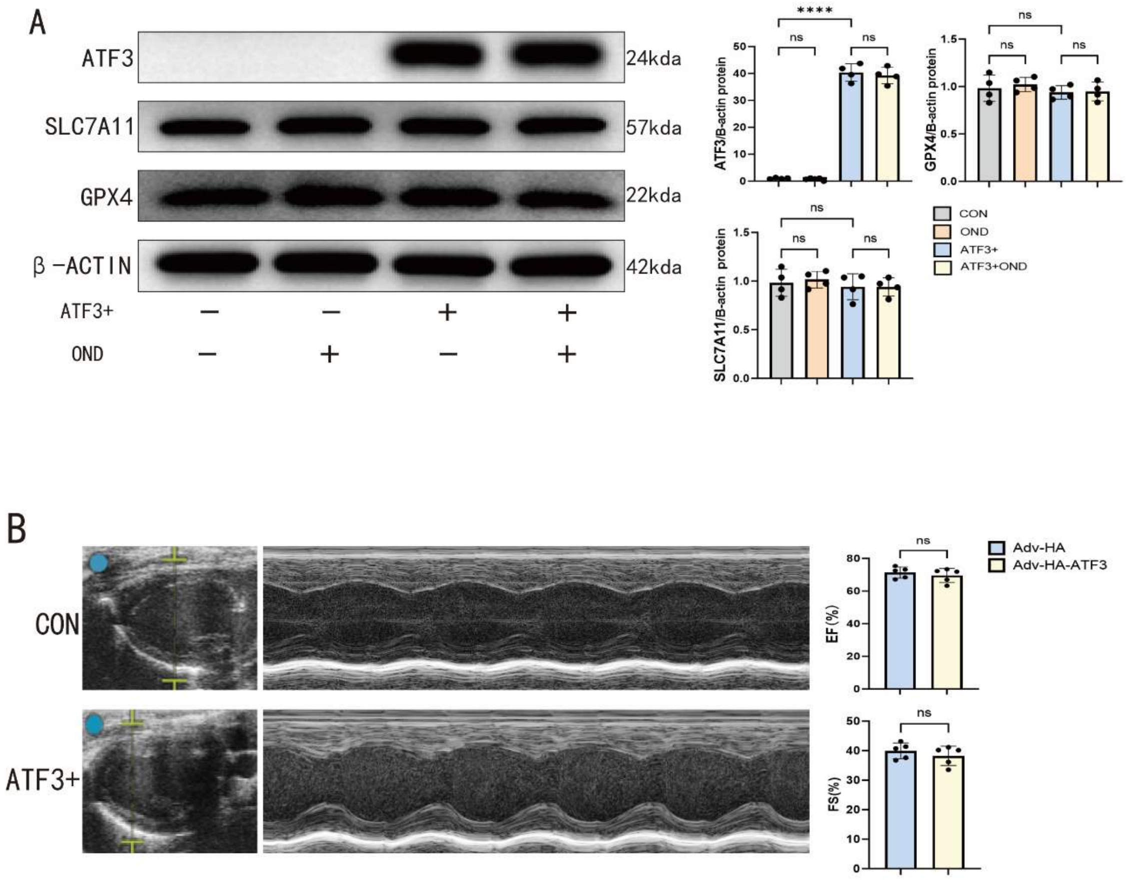

Figure S2 Specific overexpression of ATF3 in mouse cardiomyocytes under basal physiological conditions. (A) Representative western blotting bands and quantitative analysis of ATF3, SLC7A11, and GPX4 after overexpression of ATF3 and OND treatment (75uM) in NRCMs under physiological conditions. N=4. (B) Under physiological conditions, after cardiomyocyte-specific ATF3 overexpression, representative echocardiographic images and cardiac function indicators were obtained. N=5. For multiple comparisons, one-way or two-way ANOVA was used, followed by Tukey's multiple comparisons test. A p-value of less than 0.05 was considered statistically significant. The data are expressed as the mean ± standard deviation. * P<0.05, ** P<0.01, *** P<0.001, **** P<0.0001. Data were obtained from independent biological experiments.

Figure S3

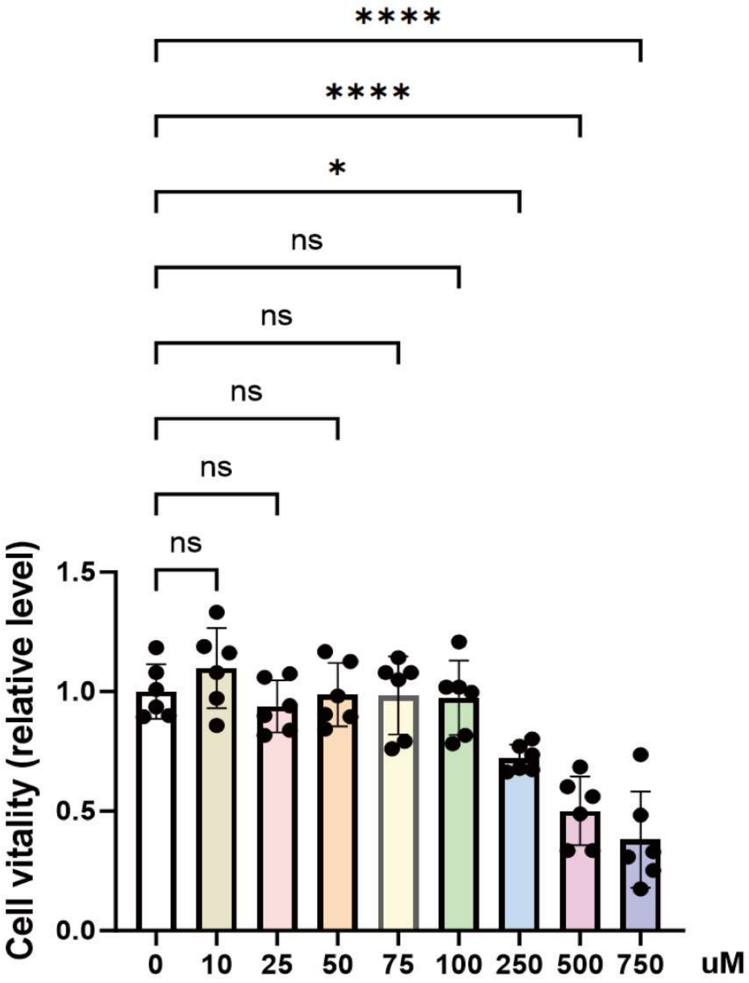

Figure S3 Effect of OND on the viability of NRCMs. NRCMs were treated with various concentrations of OND for 24 hours. Subsequently, cell viability was assessed using the CCK-8 assay kit (C0037, Beyotime, Shanghai, China) according to the manufacturer's instructions.

Table S1 Antibodies for Western blotting

Antibodies		
anti-ACSL4	selleck,F1153	1:10000
anti-PTGS2	abclonal,A25901	1:1000
anti-SLC7A11	Huabio,HA721868	1:1000
anti-GPX4	abclonal,A25009	1:5000
anti-NOX4	beyotime,AF1498	1:1000
anti-SIRT3	beyotime,AF5303	1:1000
anti-SOD2	beyotime,AG3273	1:1000
anti-AC-SOD2	selleck,F1619	1:1000
anti-OPA1	abclonal,A28496	1:1000
anti-MFN2	Huabio,ER1802-23	1:1000
anti-DRP1	Huabio,HA500487	1:1000
anti-FIS1	beyotime,AF8268	1:1000
anti-ATF3	selleck,F1121	1:1000
anti-HTR3A	proteintech,29685-1-AP	1:1000
anti-ATF6	beyotime,AF6243	1:1000
anti-GRP78	beyotime,AF0171	1:1000
anti-CHOP	beyotime,AF6684	1:1000
anti-XBP1	beyotime,AF8367	1:1000

Table S2 Primers for Real-Time PCR

	Forward	Reverse
rat-XBP1	GTCCGCAGCACTCAGACTAC	GCCCAAAAGGATATCAGACTCAG
rat-CHOP	CCACCACACCTGAAAGCAGA	AAGGTTTTTGATTCTTCCTCTTCG
rat-ATF4	TCCTTTCCTCTTCCCCTCCC	GGTTTCGTGAAGAGCGCCAT
rat-ATF6	GAACTTCGAGGCTGGGTTCA	ACTCCCAGAATTCCTACTGATGC
rat-GRP78	TTCCGAGGAACACTGTGGTG	GTCAGGGGTCGTTCACCTTC
rat-IL-1β	TCATCTTTGAAGAAGAGCCCG	TCAGACAGCACGAGGCATTT
rat-IL-6	CCAGTTGCCTTCTTGGGACT	TGCCATTGCACAACTCTTTTC
rat-IL-1α	CGCTTGAGTCGGCAAAGAAAT	GCAGTTCTCATGAAGTGAGCC
rat-TNF-α	GTAGCCCACGTCGTAGCAAA	GTGAGGAGCACGTAGTCGG
rat-ACSL4	GAGGCAGCACCTTCGATCC	AGAAGGCAGTAACGGAAGCA
rat-PTGS2	CTCAGCCATGCAGCAAATCC	GGGTGGGCTTCAGCAGTAAT
rat-ATF3	GGTGCACGGTGCTTCCC	GAAGCATCATTTTGCTCCAGTC
rat-mt-ND1	CTACATACAACTACGCAAAGGC	GCTTAGAGCTAGTGTAAGGGAG
rat-mt-ND2	TCCTAAACCCAACTATCACCAC	TGATTTTTCGTGTTTGGGTCTG
rat-mt-ND3	CCGAAAAAGCAAACCCATATGA	AGGCGATTTCTAGGTCGAATAG
rat-mt-ND4	CCATCATTCTAGACCCCCTAAC	CGTAGGCAGATTGAGCTAGTTA
rat-mt-ND5	CATGACAAAACCACGATACTCC	AGGATTGTAATGAGTAGGGCTG
rat-mt-ND6	AGTGGATGTATTGGGTGCTTAA	ACCAACTCCATAAAAAGCCCTA
rat-mt-Cytb	ATCTGCTATCCCTTACATTGGG	GTGTTAGGGTTGCTTTGTCTAC
rat-sirt3	CGGTCGTCAAGGTGGGAG	TCTGGGATACCACTGGGTGT

Table S3 Primers for Real-Time PCR

	Forward	Reverse
mus-IL-6	TGGTCTTCTGGAGTACCATAGC	GTGACTCCAGCTTATCTCTTGGT
mus-ACSL4	GGAAAGCAAACTGAAGGCGG	AATGGCCATGTCTGAAGGGG
mus-PTGS2	TGGGGGAAGAAATGTGCCAA	CAGCCATTTCCTTCTCTCCTGT
mus-TNFα	ACCCTCACACTCACAAACCAC	ACAAGGTACAACCCATCGGC
mus-ATF3	AGCGAAGACTGGAGCAAAATG	CATCCGATGGCAGAGGTGTT
mus-mt-ND1	CTAATCGCCATAGCCTTCCTAA	GTTGTTAAAGGGCGTATTGGTT
mus-mt-ND2	TTTACCCGCTACTCAACTCTAC	CATCCTATGTGGGCAATTGATG
mus-mt-ND3	CTACTTCCACTACCATGAGCAA	TGTTCATTCATATGCTAGGCCT
mus-mt-ND4	GGATCCACAGCCGTACTATAAT	TGAAGGGGGTAGAGCTAGATTA
mus-mt-ND6	GTTAGTGGGTTTGTTGGTTGTT	CCCAAGTCTCTGGATATTCCTC
mus-mt-Cytb	CCACTCATTCATTGACCTACCT	GCTCCGTTTGCGTGTATATATC
mus-IL-1α	GTCGGGAGGAGACGACTCTAA	GTTTCTGGCAACTCCTTCAGC
mus-IL-1β	TGCCACCTTTTGACAGTGATG	TGATGTGCTGCTGCGAGATT

Table S4 Antibodies for Immunofluorescence

Antibodies		
anti-ATF3	selleck,F1121	1:100
anti-4-HNE	abcam,#ab48506	1:200
anti-cTnT	abcam,#ab8295	1:200
